# Thermo‐Reversible Cellulose Micro Phase‐Separation in Mixtures of Methyltributylphosphonium Acetate and γ‐Valerolactone or DMSO

**DOI:** 10.1002/cphc.202100635

**Published:** 2022-02-25

**Authors:** Ashley J. Holding, Jingwen Xia, Michael Hummel, Harry Zwiers, Matti Leskinen, Daniel Rico del Cerro, Sami Hietala, Martin Nieger, Marianna Kemell, Jussi K. J. Helminen, Vladimir Aseyev, Heikki Tenhu, Ilkka Kilpeläinen, Alistair W. T. King

**Affiliations:** ^1^ Department of Chemistry University of Helsinki A.I. Virtasen aukio 1 00560 Helsinki Finland; ^2^ Circuvate Walderdorffstr. 4 65604 Elz Germany; ^3^ Department of Bioproducts and Biosystems Aalto University P.O. Box 11000 00076 Aalto Finland; ^4^ The Technical Research Centre of Finland (VTT) Tietotie 4e 02150 Espoo Finland

**Keywords:** UCST, ionic liquids, cellulose regeneration, spherulite, renewable resources

## Abstract

We have identified cellulose solvents, comprised of binary mixtures of molecular solvents and ionic liquids that rapidly dissolve cellulose to high concentration and show upper‐critical solution temperature (UCST)‐like thermodynamic behaviour ‐ upon cooling and micro phase‐separation to roughly spherical microparticle particle‐gel mixtures. This is a result of an entropy‐dominant process, controllable by changing temperature, with an overall exothermic regeneration step. However, the initial dissolution of cellulose in this system, from the majority cellulose I allomorph upon increasing temperature, is also exothermic. The mixtures essentially act as ‘thermo‐switchable’ gels. Upon initial dissolution and cooling, micro‐scaled spherical particles are formed, the formation onset and size of which are dependent on the presence of traces of water. Wide‐angle X‐ray scattering (WAXS) and ^13^C cross‐polarisation magic‐angle spinning (CP‐MAS) NMR spectroscopy have identified that the cellulose micro phase‐separates with no remaining cellulose I allomorph and eventually forms a proportion of the cellulose II allomorph after water washing and drying. The rheological properties of these solutions demonstrate the possibility of a new type of cellulose processing, whereby morphology can be influenced by changing temperature.

## Graphical abstract

**Cellulose spherulites** are formed during regeneration from the [P_4441_][OAc] ionic liquid and DMSO or GVL co‐solvent electrolyte mixtures, after dissolution at increased temperature and cooling of the mixtures. The thermodynamics of the initial gelation, upon cooling, support a UCST‐type transition, with additional spherulite formation occuring during the gelation process. Cellulose solvation in [P_4441_][OAc] electrolytes was also found to be highly dependent on co‐solvent polarity.

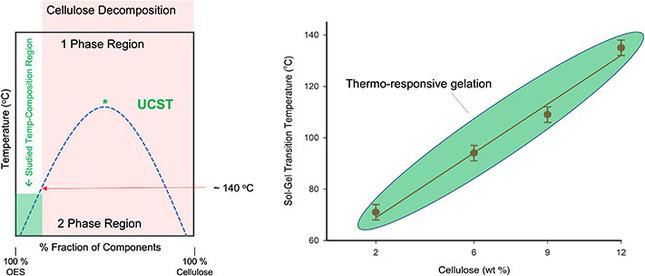

## Introduction

Mixtures of molecular solvents and ionic liquids (ILs), commonly described as ‘IL‐electrolytes’, have gained more attention recently as solvent systems for cellulose,^[1]^ biomass fractionation or pre‐treatment media,^[2]^ and for the catalytic processing of lignocellulosic biomass.^[3]^

We have previously demonstrated^[4]^ the ability of phosphonium‐based ILs, in the dissolution of cellulose, in combination with dimethylsulfoxide (DMSO) and other dipolar‐aprotic solvents. They have a high dissolution capacity when compared to other similar cellulose solvents and some structures can be phase‐separated from regeneration water, for easy recovery and recycling.^[4]^ These solutions have since been used as media for the derivatization of cellulose to produce cellulose methylcarbonates^[5]^ and for the NMR analysis of high‐molecular weight celluloses.[^6^, ^7^]

**Figure 1:**
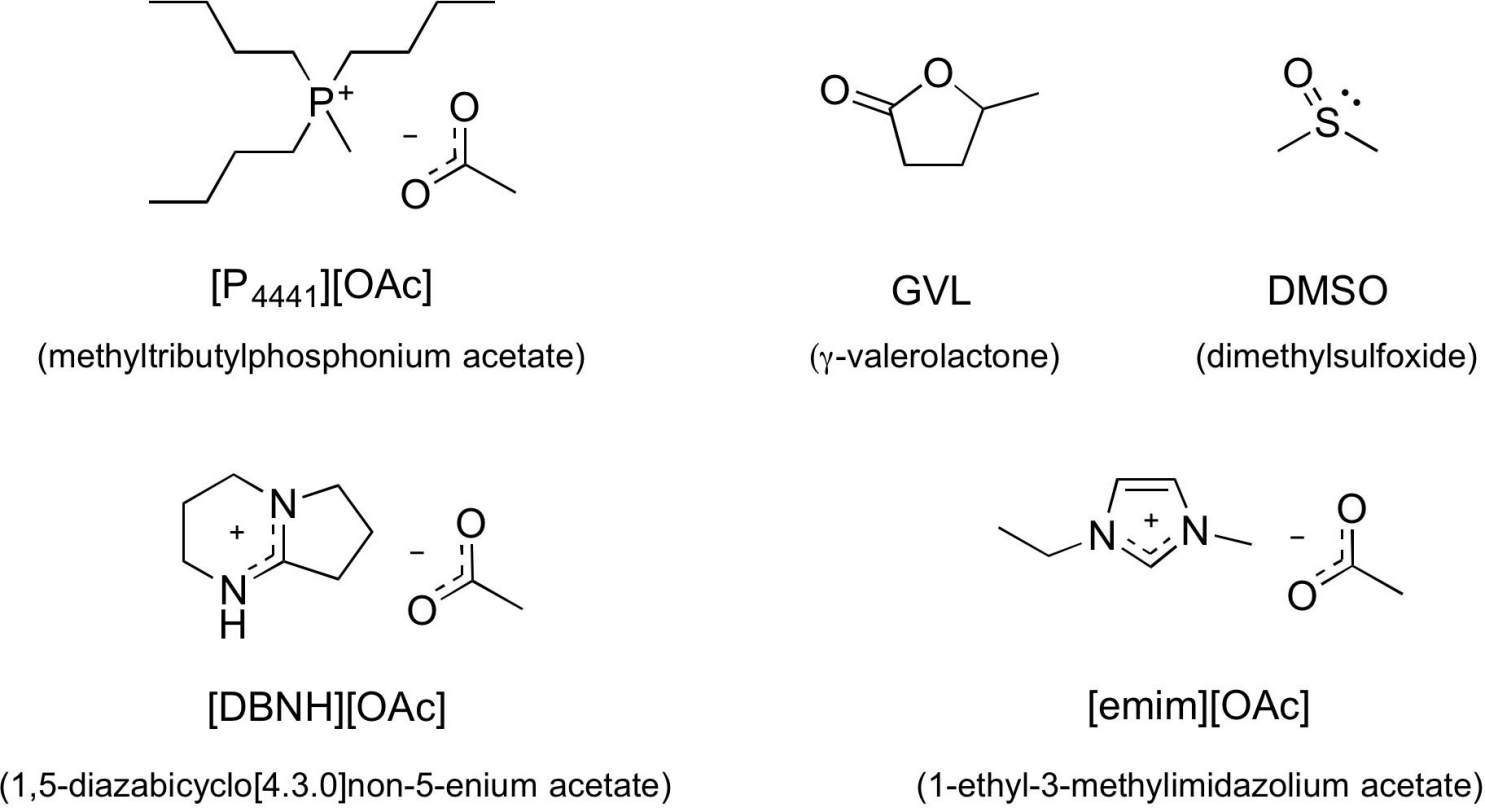
Ionic liquids and co‐solvents used in this study.

DMSO, *N,N*‐dimethylformamide (DMF) and 1,3‐dimethyl‐2‐imidazolidinone (DMI),[^1^, ^4^] are some of the most effective dipolar aprotic molecular solvents, when combined with ILs, for cellulose dissolution. However, they have a number of limitations which need to be addressed when considering large‐scale applications. DMI and DMF are toxic, and the role of DMSO as a permeator (biological solvent), is problematic. There has been much recent attention concerning the preparation and use of γ‐valerolactone (GVL) as a potentially ‘green’ media for a range of chemical processes. It is considered as a bio‐based and ‘green’ solvent, due to its supposed low toxicity,^[8]^ high boiling point and high flash point.^[9]^ In addition, GVL (Figure 1) can be synthesised *via* hydrogenation/dehydration of levulinic acid (LA), obtained from lignocellulosic biomass.

Phosphonium ionic liquids have broad chemical stabilities, high thermal stabilities and are excellent cellulose solvents, as their dipolar aprotic solvent organic electrolyte solutions (OESs). Therefore, we focus mainly upon the phosphonium IL methyltributylphosphonium acetate, [P_4441_][OAc] (Figure 1), as a relatively low‐toxicity structure which is simple to prepare in the laboratory. Compared to 1‐ethyl‐3‐imidazolium acetate [emim][OAc] (Figure 1), a highly effective room temperature IL for cellulose dissolution, and 1,5‐diazabicyclo[4.3.0]non‐5‐enium acetate [DBNH][OAc] (Figure 1), a distillable superbase IL[^10^, ^11^] applied as solvent in fibre spinning processes,^[12]^ it has also a relatively low toxicity against several cell lines, especially in comparison to its longer chain homologues.^[13]^ The noticeably lower toxicity of [P_4441_][OAc] avoids some of the main limitations of other phosphonium ILs whilst maintaining their beneficial features. However, due to the reduced hydrophobicity, these structures are only phase‐separable from water with the addition of kosmotropic salts.^[4]^ Therefore, we would like to present our preliminary results concerning the phase‐behaviour of these cellulose‐ionic liquid solutions upon cooling; emphasis is put on understanding the mechanism and thermodynamics, for the purposes of the potential of this phenomenon as a novel method of controling cellulose regeneration.

## Experimental

### Materials

MCC (Microcrystalline Cellulose DP_n_: 87.03, DP_w_: 356.94, PDI: 4.14) was purchased from Sigma Aldrich Ltd, Enocell Birch Pre‐Hydrolysis Kraft (PHK) Pulp was provided by Stora Enso (Uimaharju, Finland, DP_n_: 468, DP_w_: 1560, PDI: 3.33). Tributylmethylphosphonium methyl carbonate ([P_4441_][MeCO_3_]) solution (80 % in methanol) was purchased from Iolitec GmbH. Glacial acetic acid (>98.5 wt%), was purchased from Sigma Aldrich (Finland). Reichardt's Dye (2,6‐Diphenyl‐4‐(2,4,6‐triphenyl‐1‐pyridinio)phenolate), *N,N*‐diethylnitroaniline and 4‐nitroaniline dyes for Kamlet‐Taft measurements were purchased from Sigma Aldrich (Finland). 1‐ethyl‐3‐imdazolium acetate ([emim][OAc], >98 %) was purchased from Iolitec GmbH (Heilbronn, Germany).

### Synthesis of [P_4441_][OAc]

[P_4441_][OAc] was synthesised from [P_4441_][MeCO_3_] (80 wt% in methanol) by metathesis reaction, with the addition of one equivalent of acetic acid and eventual evaporation to dryness. Compound identification, assignments and purity were determined through ATR‐IR, NMR (^1^H, ^13^C & HSQC), DSC (mp=41 °C) and water content was monitored to be ∼0.2 wt% by Karl‐Fischer analysis, for the initial synthesis. WAXS analysis was performed on the ionic liquid which had crystallised from the melt. Single‐crystal X‐ray structure determination was also performed on larger crystals that had crystallised around the top of the solid paste. Full experimental and characterisation is given in the Supporting Information.

### Observation of the Phase‐Separation Phenomenon

The cloud point temperature (*T*_cp_) defined the boundary of phase‐separation upon cooling in cellulose solutions of [P_4441_][OAc]:GVL (70 : 30 w/w). *T*_cp_ was determined by means of light transmittance measurements at a wavelength of 600 nm using a UV–Vis spectrophotometer (Jasco V‐750). Quartz cuvettes used for measurements were two side polished with path‐length of 10 mm (Hellma Analytics). The baseline of UV‐spectrometer was corrected with distilled water at RT. Then we pre‐heated the spectrophotometer to 90 °C, prior to the measurements. Cellulose solutions with concentration varying from 7 to 9.5 wt% have been examined. 2 ml MCC solutions were prepared in 10 ml vial with magnetic stirring at 120 °C for 5 min. Then the solutions were quickly transferred to the cuvettes and sealed with Teflon tape. The cuvettes were kept at 120 °C for 1 min more then quickly transferred to the UV‐spectrometer. These were then equilibrated at a cuvette temperature of 90 °C for 10 min. Transmittance was measured with a cooling rate of set 1 °C/min in the range of 90–10 °C.

### Phase Diagram Dissolution Experiments

Dissolution experiments were conducted in a similar manner to our previous studies,^[2]^ with increments of cellulose (MCC or Enocell Pre‐Hydrolysis Kraft Pulp) being sequentially dissolved in a pre‐mixed IL‐co‐solvent solution at 100 °C until a cloud point is reached. When a cloud point was reached, the temperature was increased to 120 °C, for a short period, to test if further material would dissolve, at the desired saturation temperature. The upper boundary is taken as the midpoint between the cloud point and the last addition of cellulose, where each addition is ca. 0.5 wt% of the total mass of the mixture.

### Preparation of Regenerated Micro Particles

Microparticles were prepared for microscopy, WAXS, ^13^C CP MAS NMR and SEM analysis to attempt to better characterise the crystallinity and morphology of the regenerated particles. This was achieved by dissolving the samples, according to the method above. The samples were then cooled to micro phase‐separate the cellulose in the IL‐electrolyte solution, for microscopy and WAXS analysis. GVL was then removed in a vacuum oven to produce a sample also for WAXS and SEM analysis. [P_4441_][OAc] was then removed from this mixture by sonication with acetone. Sonication was required to disaggregate the microparticles to remove almost all ionic liquid. These were then analysed by WAXS and ^13^C CP MAS NMR. The full conditions for the regeneration and analyses are given in the Supporting Information.

### Additional Analyses

Viscoelastic properties of key cellulose‐IL‐GVL mixtures were measured using rheometry. This included measuring temperature sweep measurements in the linear viscoelastic regime. This allowed for determination of the temperature and concentration ranges where the sol‐gel transition occurs. Kamlet‐Taft parameterisation was performed over different compositions of [P_4441_][OAc] and dipolar aprotic solvent (GVL and DMSO) to give an indication of what properties of the solvent are required to induce the thermo‐responsive behaviour. Differential scanning calorimetry (DSC) was measured for some higher concentration cellulose solutions, to determine if the phase‐changes were endothermic or exothermic. Full details are in the Supporting Information.

WAXS analysis was performed in Bragg‐Brentano (reflectance) geometry using a Rigaku SmartLab or PANalytical X'Pert Pro MPD X‐ray diffractometer. Crystallite sizes were calculated using the Scherrer equation [Eq. 1]:^[14]^


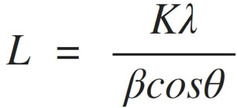




which is used for determining the lateral dimension (L) of cellulose crystallites, where *β* is the full width at half maximum (FWHM) for diffraction peaks, K (the Scherrer constant) is 0.94 for spherical crystallites with cubic symmetry. This is applied for determination of cellulose crystallite size.^[15]^ *λ* is the X‐ray wavelength (1.54 Å in our case for Cu K*α*) and *θ* is 2*θ*/2 (in radians). The FWHM values were determined by applying Gaussian functionals, in ‘*Fityk*’,^[16]^ to the sharper peaks labelled in the diffractograms. These were chosen as the most prominent peaks in the diffractograms and most likely to give accurate crystallite sizes.

## Results and Discussion

### Thermo‐reversible Phase‐Transition Phenomenon

**Figure 2:**
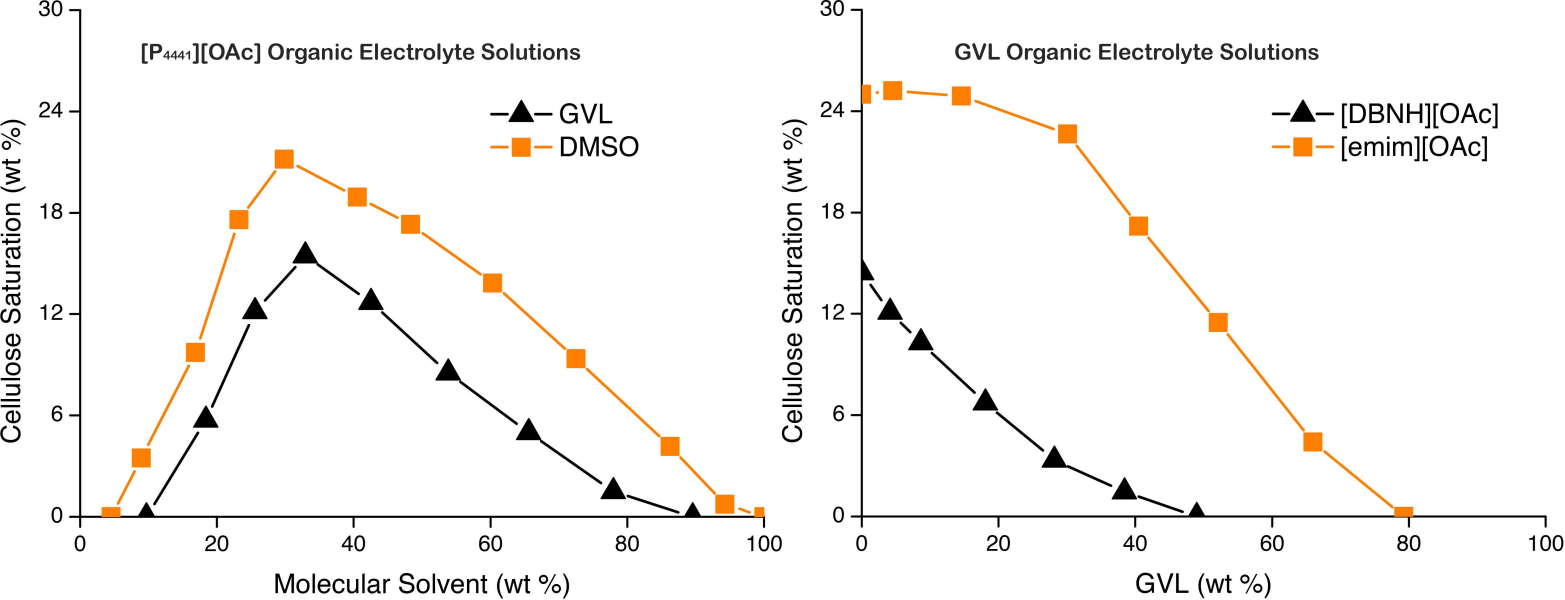
Cellulose solubility diagram showing the saturation points of cellulose (birch pre‐hydrolysis kraft pulp) at maximum of 120 °C in a) [P_4441_][OAc] with different concentrations of GVL and DMSO and b) [emim][OAc] and [DBNH][OAc] with different concentrations of GVL.

A preliminary examination of the dissolution of cellulose in solutions of [P_4441_][OAc] and DMSO, or GVL, was initially conducted (Figure 2a), by measuring the saturation points at 120 °C. This was compared to the saturation of cellulose in the well‐known cellulose dissolving ionic liquids [emim][OAc] and [DBNH][OAc], as their GVL electrolytes (Figure 2b). [emim][OAc] clearly has the best ability to dissolve cellulose, when the saturation points are compared using GVL. The response of [emim][OAc] with GVL is close to that of DMSO presented in our previous study,^[4]^ except that the solubility drop‐off occurs a little sooner as GVL composition increases. [DBNH][OAc]‐GVL compositions are, comparatively, rather poor at dissolving cellulose. Pure [DBNH][OAc] dissolves around 15 wt% PHK pulp, which is sufficient for Lyocell‐type fibre‐spinning. Maximum dissolution capabilities start at the pure ionic liquids for both [emim][OAc] (25 wt%) and [DBNH][OAc] (15 wt%). The latter value is comparable to the 16 wt% quoted for PHK pulp saturation in [DBNH][CO_2_Et] (propionate) published previously.^[10]^ However, it has been possible to prepare dopes and spin fibres, of high quality, using 17 wt% dissolving pulp in [DBNH][OAc].^[12]^

By contrast, pure [P_4441_][OAc] does not dissolve cellulose, as was reported previously for the long‐chain phosphonium IL homologues.^[4]^ When mixed with DMSO or GVL, the optimum co‐solvent amount is ∼30 wt%. DMSO allows for higher saturation with cellulose compared to GVL, which is not surprising as DMSO is a highly effective dipolar aprotic solvent. However, the optimum cellulose saturation points in the GVL are still rather high.

What is most interesting is that for [P_4441_][OAc] and either DMSO or GVL, at lower temperatures (<50 °C), solutions containing more than 50 wt% [P_4441_][OAc] did not rapidly dissolve cellulose, at temperatures close to, or just above, room temperature. This is in contrast to the compositions of 50–80 wt% [P_4441_][OAc] or the DMSO electrolytes of longer‐chain phosphonium acetates, where dissolution typically starts even at room temperature, especially for the DMSO‐based OESs.[^4^, ^6^] For the samples with >50 wt% [P_4441_][OAc] close to the saturation points, dissolution only occurred when heating to temperatures approaching and above 100 °C. Initial indications seemed to show that the required temperature for complete dissolution increased with the addition of cellulose, which was especially pronounced in the region of lowest molecular solvent composition. Upon cooling, these solutions became turbid (after sitting for several hours) indicating a strong decrease in solution quality resulting in partial micro phase‐separation of the cellulose component. Upon increase in temperature again, the samples were observed to redissolve and the system could be cycled, indicating thermos‐reversible behaviour.

Both [emim][OAc] and [DBNH][OAc] also appeared to show thermo‐reversible behaviour with GVL but not with DMSO (just above the saturation points), unlike [P_4441_][OAc] in which this behaviour is observed for both DMSO and GVL. This suggests the thermodynamic quality of the [P_4441_][OAc]:GVL solutions are decreased when compared to these more protic ILs and DMSO electrolytes, respectively. Thermo‐reversible phase‐separation of [emim][OAc]:1,3‐dimethyl‐imidazolidinone (DMI):MCC mixtures has also previously been observed.[^17^, ^18^] In this case, a decrease in DMI composition towards the cellulose‐dissolving pure [emim][OAc], results in a decrease in the phase‐transition temperature, as opposite to what is observed with [P_4441_][OAc]:GVL. The phase‐transition temperature and kinetics of separation are also rather defined, as opposed to [P_4441_][OAc]:GVL, indicating a much more robust system for thermo‐reversible phase‐separation. Moreover, IL:DMI:MCC solutions seem to readily segregate upon cooling into distinct IL‐ and cellulose‐enriched lower and DMI‐enriched upper liquid layers.[^17^, ^18^] Whereas, the [P_4441_][OAc]:GVL:MCC system forms gel which does not segregate, presumably due to gel‐formation.

### Sol‐Gel Viscoelastic Properties and Cloud Point

To understand the potential applicability of this system as a tool for production of novel materials, oscillatory shear rheological measurements were used to evaluate the temperature response of the cellulose solutions based on [P_4441_][OAc] and GVL with Enocell PHK pulp, in more detail. GVL may be of more interest than DMSO as it does not suffer from certain hazards that DMSO is well known for, *i. e*. biological solvent properties. It is also potentially bio‐based,^[8]^ has a low vapour pressure and high flash point.^[9]^ However, it is currently quite expensive.

**Figure 3:**
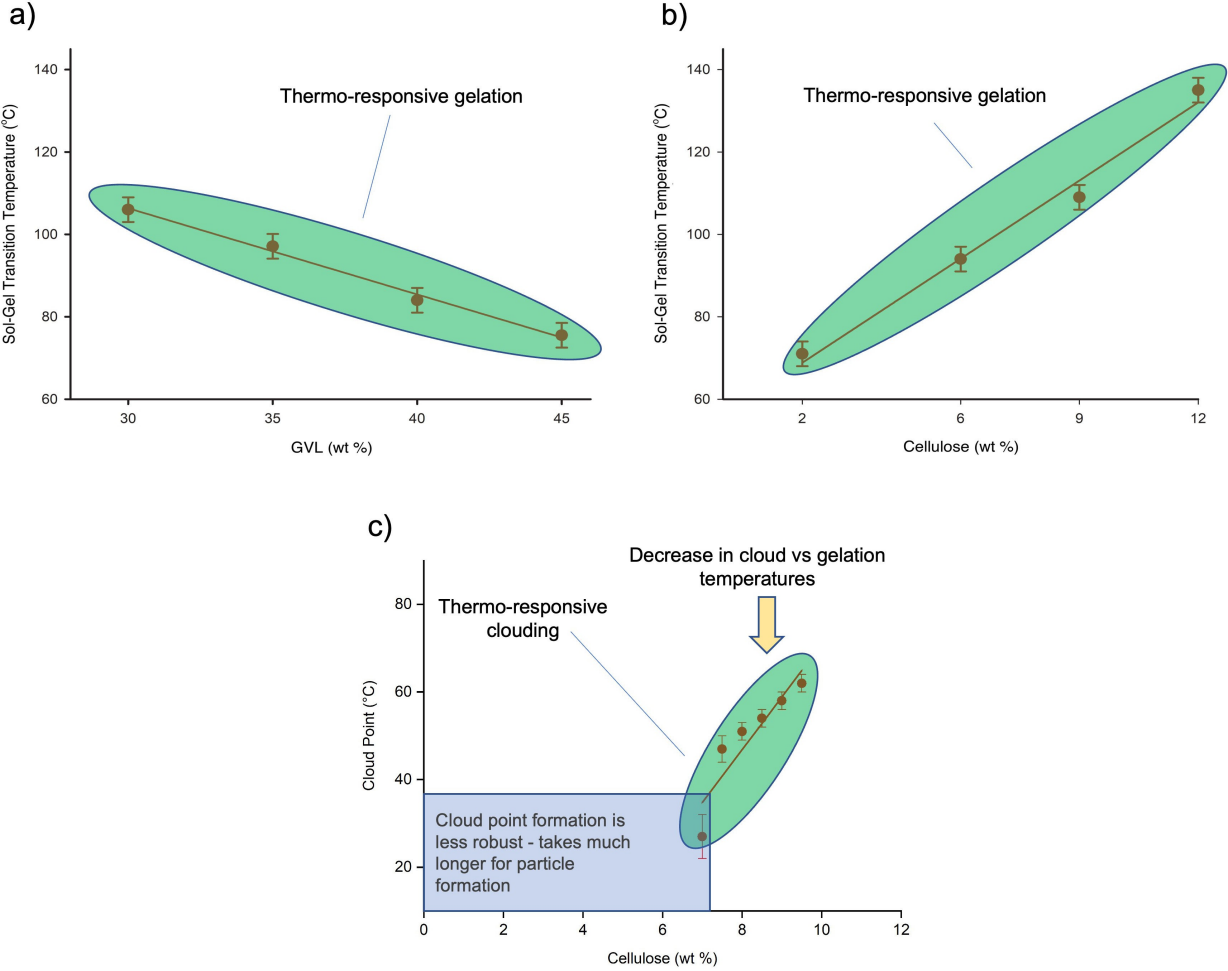
Sol‐Gel Transition temperature for a) different compositions of [P_4441_][OAc]:GVL for 9 wt% PHK pulp and b) different concentrations of PHK pulp in [P_4441_][OAc]:GVL (70 : 30 w/w). c) The phase diagram for MCC in [P_4441_][OAc]:GVL (70 : 30 w/w) (*T*_cp_ is determined by measuring light transmittance using a UV‐vis spectrophotometer).

The storage, and loss moduli, and complex viscosity of the cellulose solutions in the thermo‐responsive region of GVL and [P_4441_][OAc] were recorded through a dynamic temperature sweep, consisting of a heating and cooling phase. When passing from the state of being a poor, non‐dissolving solvent to a good solvent, we would expect to see a distinct change in the storage and loss moduli, indicating a transition from a gel to a sol (solution) phase. This so‐called ‘Sol‐Gel transition’ temperature (SGTT) should give an indication of the onset of the phase change in the solutions. Several methods have been proposed to determine the sol‐gel transition temperature from the dynamic moduli.[^19^, ^20^, ^21^, ^22^] Herein, we have defined the SGTT as the crossover of the storage and loss modulus (Supporting Information) in the temperature sweep, as was demonstrated to be suitable for cellulose‐IL systems previously.^[21]^ After studying both changes in wide cellulose concentration (2–12 wt%) for a fixed GVL composition (30 wt% in pure OES) and changes in GVL (30–45 wt% in pure OES) for a fixed cellulose concentration (9 wt%), SGTT is observed to increase as a function of cellulose concentration, for 30 wt% GVL solutions (Figure 3a), and GVL concentration, for 9 wt% cellulose solutions (Figure 3b). Higher concentrations of cellulose or variation outside the GVL compositions, shown in Figure 3, do not give reliable results due to a drop off in solubility or too high transition temperatures, as we are now already in the poor solvation regimes (Supporting Information). Thus, for this system the optimum phase‐transition temperature, avoiding thermal decomposition of the cellulose, is around 9 wt% (*T*_SGTT_=75–110 °C). This concentration is relatively low but there is clearly room for optimisation, depending on co‐solvent choice, cellulose substrate and regeneration conditions. As [P_4441_][OAc]:DMSO OESs are even better at solvating cellulose, over these compositions, it is likely that the accessible concentrations can be pushed near to 15 wt%, yet, maintaining relatively low transition temperatures. This should be studied more thoroughly in further work.

The cloud point temperature (*T*_cp_) defines the point at which highly light‐scattering particles of cellulose are formed in the mixture. When we investigate the phase separation of cellulose from [P_4441_][OAc]:GVL (70 : 30 w/w) upon cooling, the *T*_cp_ can be determined by change in light transmittance, using UV‐vis spectrometry (Figure 3c). The transmittance changes of the cellulose solutions, as a function of temperature, are shown in Figure S31 in the Supporting Information (SI). MCC was used to allow for more rapid particle formation for this study, which is thought not to affect the thermodynamics of the phase‐transition phenomena but rather the kinetics, due to the lower molecular weight (lower viscosities). The cellulose concentrations we were able to measure are between 7.5 wt % to 9.5 wt %. Below 7.5 wt %, the phase separation kinetics became very slow and *T*_cp_ cannot be defined. This is demonstrated in Figure S31 in SI for solution of 7 wt % and corresponding large experimental error in Figure 3c. The cellulose concentration region below 7.5 wt % is marked as a transparent blue square in Figure 3c. In this region, 1 °C/min cooling steps resulted in no immediate clouding of the solutions. However, after equilibration at RT (20 °C) for a longer period (several hours), the UV turbidity eventually increases as scattering particles are formed. This suggests that *T*_cp_ is not only influenced by the initial solution compositions but also by the cooling conditions and below certain concentrations the measurements contain increasing error. When the concentration of cellulose is above 9.5 wt%, the system becomes too viscous and cellulose dissolution at 120 °C takes a much longer time. Annealing colourises the mixture and the transmittance cannot be measured accurately.

What is interesting is that the cloud points are significantly lower than the SGTTs. Gel‐formation seems to occur initially, as temperature decreases, with clouding only occurring at even lower temperatures (in lower mass transfer regimes), and after some time. This may be one reason why this system is not as robust as those previously studied by Clough^[17]^ and Xia.^[40]^ This points the way towards careful selection of IL and co‐solvent, to develop more robust systems. While the rheology and UV measurements were performed using different molecular weight high‐purity celluloses (PHK pulp and MCC), the temperature onset of gelation is expected to be the same but the effect of gelation will more noticable for the higher molecular weight PHK pulp. For the clouding of the sample, the lower molecular weight MCC offers more accurate (elevated) clouding temperatures, due to the increased mass transport that the lower molecular weight offers.

### Phase‐Transition Thermodynamics

**Figure 4:**
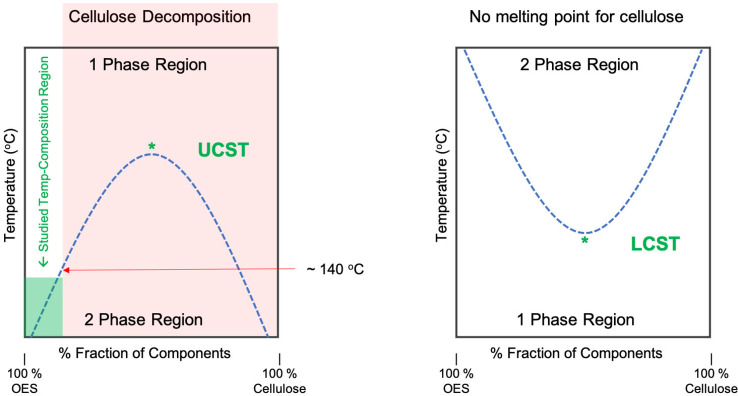
Idealized definition of UCST and LCST in relation to cellulose dissolution in organic electrolyte solutions. The red arrow points to the saturation point where cellulose degradation becomes significantly accelerated under these solvent conditions.

When two non‐identical components (A & B) have a mixing temperature, above which they are miscible over the whole composition of A and B (and non‐miscible below that temperature), they are said to have an upper critical solution temperature’ (UCST, Figure 4). The dissolution at the UCST or other critical solution temperatures, along the phase‐composition, are not due to a kinetic effect where increase in temperature induces a faster dissolution, but due to overcoming a mechanistic activation barrier. This transition is thermodynamic and should be reversible.

Lower critical solution temperature (LCST)‐like thermo‐responsive behaviour is also observed for aqueous NaOH‐based solvents and some organic electrolyte cellulose solutions. Sobue *et al*.^[23]^ have demonstrated a region in their NaOH wt %‐temperature phase‐diagram marked as ‘Q’ (German: quellen, English: swollen) which seems to be a liquid‐like transit point between cellulose crystalline allomorphs containing parallel and anti‐parallel chains. This was only accessible over a defined NaOH (aq) concentration range and only after cooling below −6 °C. Budtova and Navard have reviewed similar aqueous NaOH‐based systems and the requirement for low temperature, to facilitate cellulose dissolution, seems to be a feature of all these systems. However, the systems are not thermo‐reversible due to a progressive gelation that occurs, which is highly temperature dependent and typically results in an increasing composition of cellulose II upon complete regeneration.^[24]^

From the strict IUPAC Goldbook definition,^[25]^ the terminology of LCST or UCST requires critical temperatures showing phase miscibility over the whole phase composition. Therefore, in the case of cellulose, it is practically impossible to determine if these examples can be defined as showing LCST or UCST behaviour (Figure 4). Cellulose is typically a porous solid and does not have a measurable melting point, so high cellulose compositions will never reach a pure liquid state. This is one limitation of the definition, which dates back to a book titled ‘Alloy Phase Equilibrium’ by Alan Prince in 1966.^[26]^ In our case, where we observe thermo‐reversible gelation, we also have the problem of not being able to measure the actual UCST, as above about 15 wt % cellulose, the phase‐transition temperature rises above ∼140 °C, which starts to rapidly decompose cellulose (Figure 4). While the definitions for UCST and LCST cannot be applied to these cases cleanly, they are conceptually rigorous and appropriate for the wider scope of applications. The question remains, does this SGTT behaviour follow UCST‐type thermodynamics?

**Figure 5:**
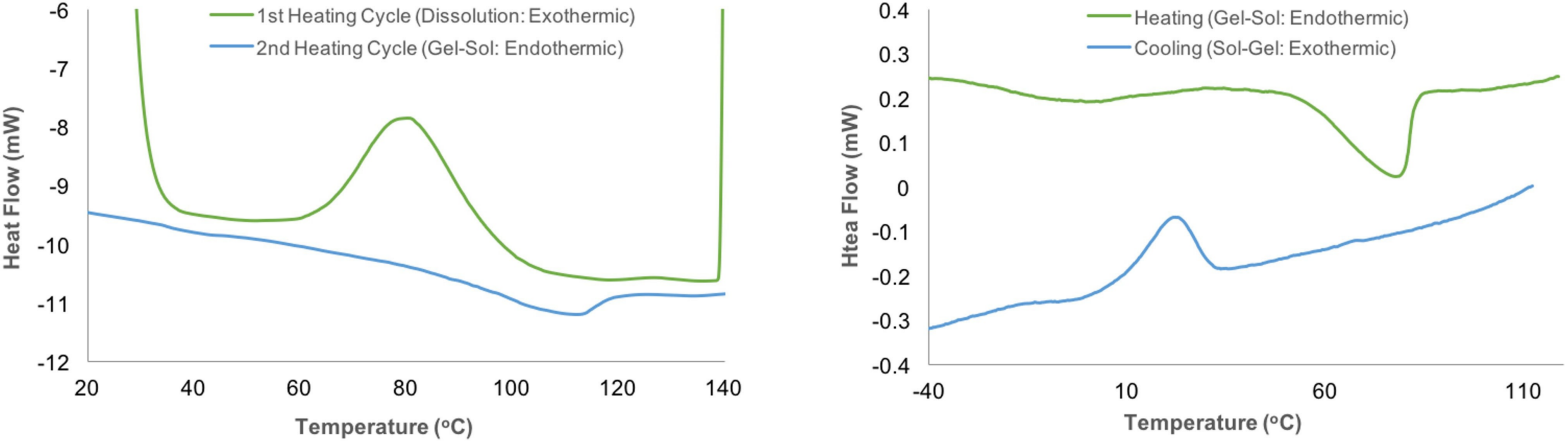
DSC of a) 12 wt% MCC in [P_4441_][OAc]:GVL (70 : 30 w/w) from frozen to observe the sign of the initial exothermic ‘dissolution’ enthalpy and b) 20 wt% MCC in [P_4441_][OAc]:DMSO (80 : 20 w/w) to observe the reversible endothermic gel‐sol transition, after exothermic dissolution and cooling.

To determine if we have a typical enthalpy‐driven dissolution process (ΔH is dominating the ΔG), or actual UCST‐type thermodynamics (TΔS is the same as ΔH at the phase‐transition), DSC was performed (Figure 5) on two samples: 1) 12 wt % MCC in [P_4441_][OAc]:GVL (70 : 30 w/w), and 2) 20 wt% MCC in [P_4441_][OAc]:DMSO (80 : 20 w/w). After trial and error, these compositions were chosen to illustrate the thermodynamics of the phase‐transitions. Otherwise, the heat‐flows were not significant at low concentrations and repetitions were not very reproducible. With the [P_4441_][OAc]:GVL sample, the cellulose was dispersed onto the ice‐chilled OES. The DSC was run from −40 to 140 °C and cycled once. This was to determine the enthalpy sign upon initial dissolution (1^st^ heating cycle) from the cellulose I microcrystalline state (hence measuring from the unmixed frozen state) and the enthalpy sign after regeneration and redissolution (2^nd^ heating cycle). What we find is that the initial ‘dissolution’ is exothermic, consistent with recent reports for cellobiose^[27]^ and for cellulose.^[28]^ However, the 2^nd^ heating cycle shows an endothermic phase transition, which is consistent with UCST‐type thermodynamics (endothermic dissolution). With the [P_4441_][OAc]:DMSO sample, we could not reproduce these results as the DMSO OES is a much better solvent, leading to more rapid swelling and dissolution. Consequently, the samples would already significantly swell before the initial equilibration steps, for the DSC measurement. Therefore, in this case we measured the phase‐transition only from the gel to the solution state. We measured heating and cooling cycles, after an initial swelling‐dissolution and cooling to below room temperature. When heated and cooled, we observed an endothermic gel‐sol transition, followed by an exothermic sol‐gel transition upon cooling.

To dissolve a polymer in solvent, the Gibbs free energy of mixing must be negative. Considering the Gibbs equation, ΔG_mix_=ΔH_mix_−TΔS_mix_, a mixture displaying a UCST would always have a positive (endothermic) enthalpy of mixing, ΔH_mix_, and entropy of mixing, ΔS_mix_, in order for the Gibbs free energy of mixing, ΔG_mix_, to become negative, as the temperature increases. The ‘dissolution’ process of cellulose in some ionic liquids has been studied previously with DSC calorimetry and are all exothermic upon ‘dissolution’.^[27]^ Much more accurate studies have been performed for cellobiose in ionic liquid electrolytes. These also show negative enthalpies upon, dissolution, for many good solvents and positive enthalpies for worse solvent mixtures, depending on the concentration.[^28^, ^29^]

In addition, rheology of some ionic liquid‐based solvents for cellulose have been studied where the thermodynamic quality of the solvent is said to decrease with an increase in temperature.[^29^, ^30^] In this case, we have the opposite effect, where the thermodynamic quality of the solution increases with temperature, from a poor solvent to a good solvent. As a polymer solution moves past the so‐called theta (θ) point, the solvent becomes “poor” when polymer‐polymer interactions start to dominate, rather than polymer‐solvent. This is clear UCST‐type thermodynamic behaviour, even if it may not fully meet the definition for UCST behaviour.

### Mechanism and Parameters of Solvation

Solvent hydrogen‐bond acidity and basicity^[10]^ are evidently important parameters in understanding the dissolution of cellulose. Typically employed to characterise solvation interactions, these can be represented by solvochromatic solvent parameter scales, such as the one proposed by Kamlet and Taft.^[31]^ High hydrogen‐bond basicity is demonstrably a minimum criterion, for a potential non‐derivatising cellulose solvent. However, other factors (related to amphiphilicity) are also clearly important and rarely discussed in the ionic liquid literature.

**Figure 6:**
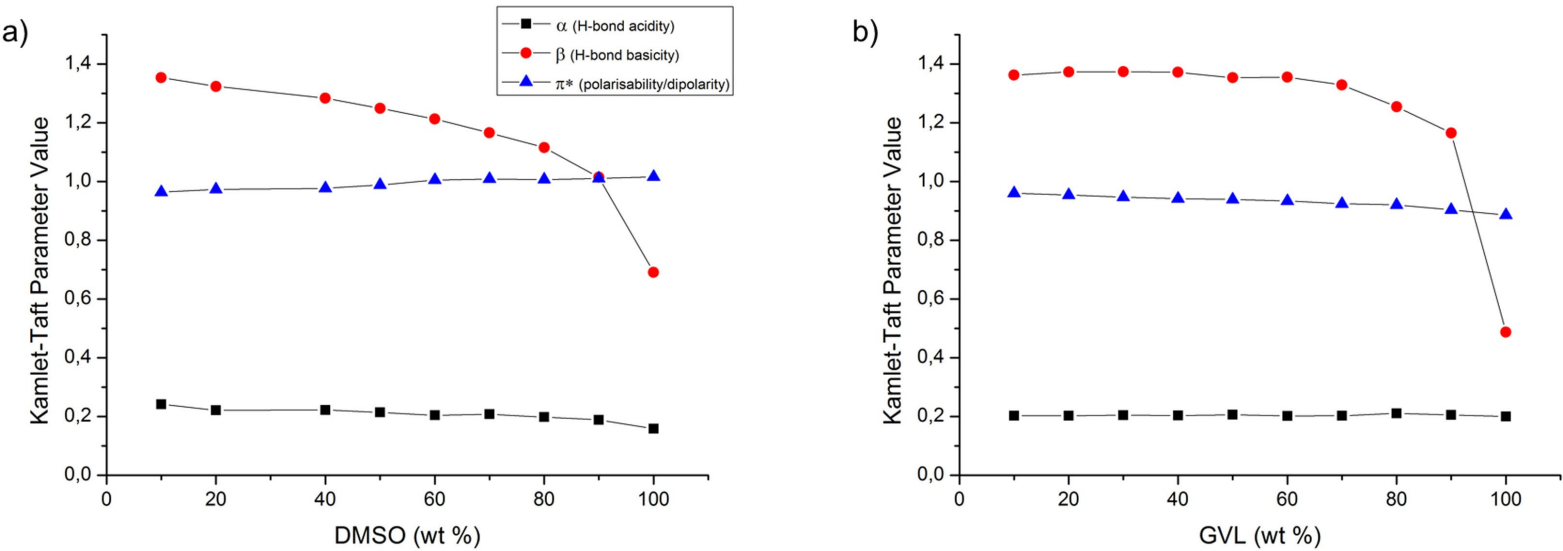
Kamlet‐Taft parameters for all compositions of [P_4441_][OAc] and either DMSO (a) or GVL (b).

There is no clear correlation between the Kamlet‐Taft *β* (hydrogen‐bond basicity) and the dissolution capacity of cellulose in these mixtures, for both GVL and DMSO (Figure 6), over the whole composition range; *β* values remain high over the whole composition at least until close to pure co‐solvent. The lower cellulose solubility at the high IL concentrations is also not echoed by the *β* values, having their highest values approaching the pure ionic liquid. There is obviously a clear drop‐off in solubility correlating with a decrease in *β*, approaching the pure molecular solvents. While the *β* values for the DMSO and GVL compositions steadily decrease, both the acidity (*α*) and dipolarity/polarizability (π*) parameters stay quite steady over the whole solvent ranges.

**Figure 7:**
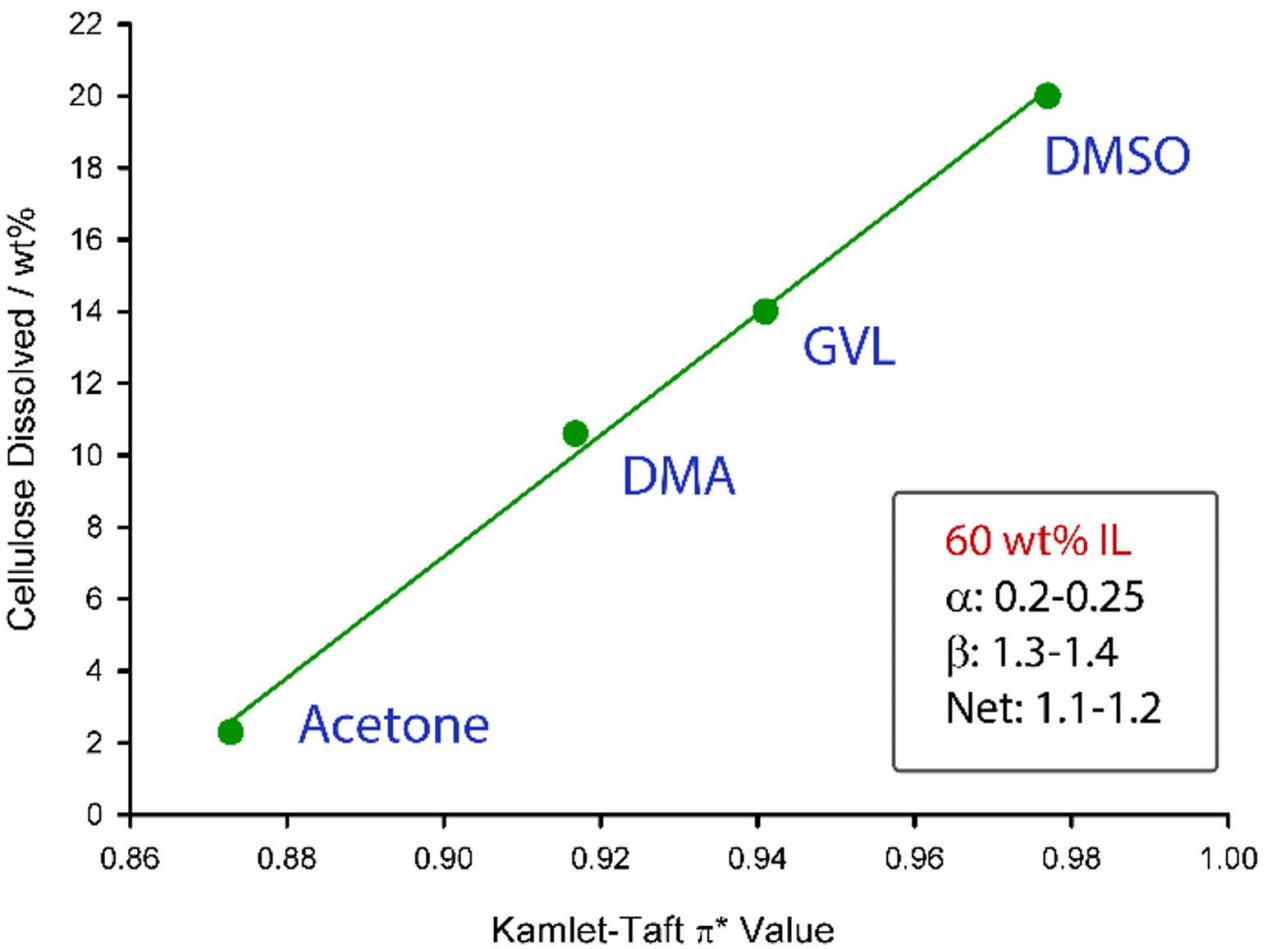
Variation in the PHK pulp saturation points at the ∼ maximum saturation OES composition of [P_4441_][OAc]:co‐solvent (70 : 30 w/w), *vs* either Acetone, DMA, GVL or DMSO as co‐solvent.

However, by comparing the same ionic liquid composition (60 wt%) in in electrolytes containing acetone, DMA, GVL, and DMSO, the cellulose saturation points seem to correlate strongly with the π* value of the electrolytes themselves (Figure 7).

Sufficient ion‐pair separation has previously been proposed to be a requirement for increased interaction with cellulose I crystallites, leading to solubility, in a molecular dynamics study into homologues of dialkylimidazolium chlorides.^[32]^ A further experimental study has confirmed increased mobility of anion and cation, through conductivity measurements, after solvation of dialkylimidazolium acetates in DMSO.^[33]^ Moreover, it was shown that the conductivity, as a function of DMSO composition, showed a strong correlation with the cellulose solubility, for the same DMSO compositions. This suggests that the more effective the ability of the co‐solvent to solvate the anion and cation, the greater is the capacity for cellulose dissolution. Therefore, dipolar aprotic solvents work best as co‐solvents for cellulose, with an increasing degree of ion‐pair separation, allowing for stronger interactions with cellulose. It may be that with phosphonium ILs, the low electron dispersity cation, with a strong formal point charge expected to lie on the phosphorous atom, leads to a strong ion‐pair interaction. This interaction is likely stabilised in the presence of strongly dipolar aprotic solvents, without significantly reducing the Hydrogen‐bond basicity, as would be expected with Hydrogen‐bond donating polar protic solvents.

### Crystal Structure and Acidity of [P_4441_][OAc]

**Figure 8:**
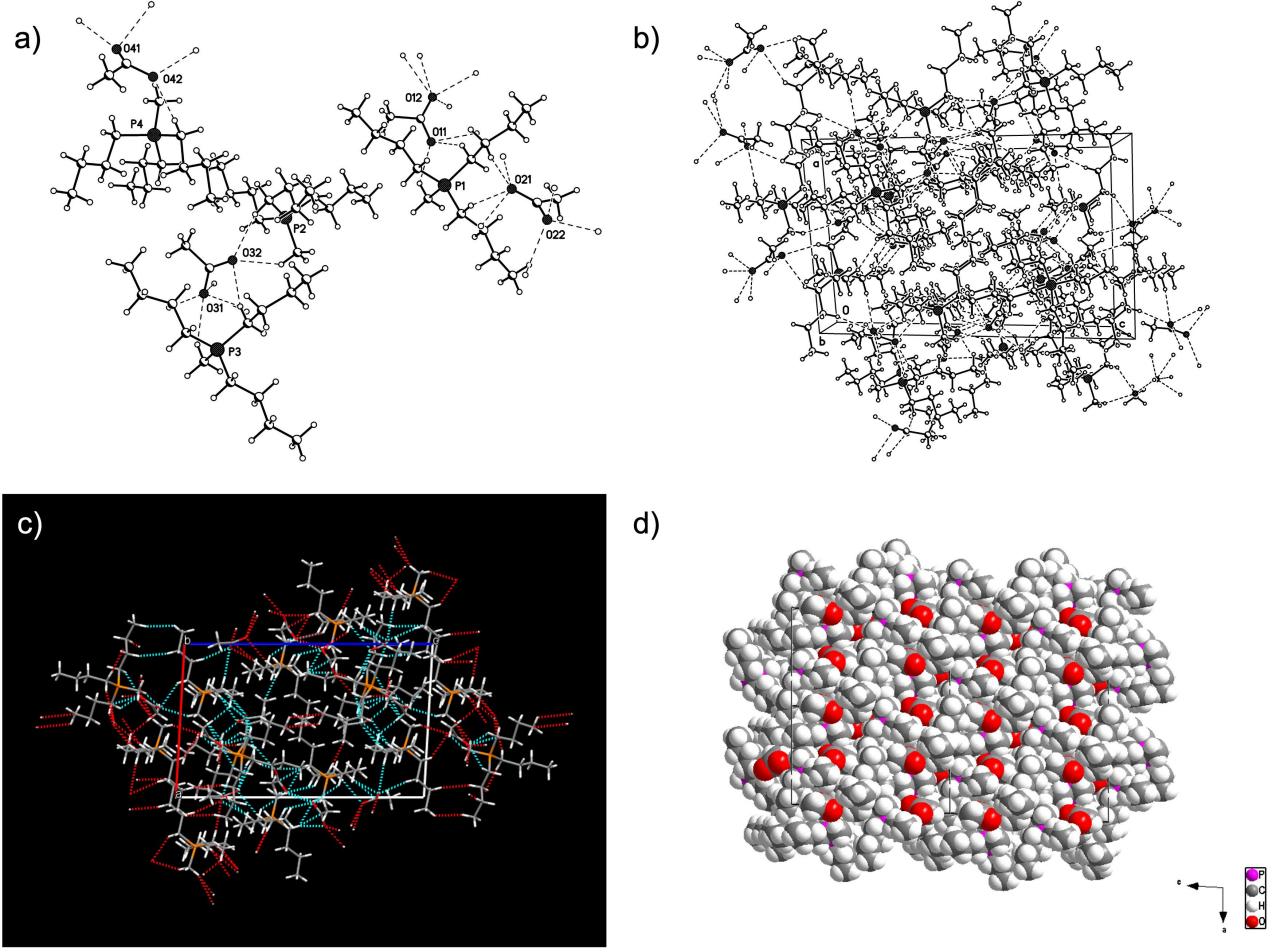
X‐Ray Structure for [P_4441_][OAc]: a) Crystallographic independent formula moieties of [P_4441_][OAc] in the asymmetric unit showing the weak CH⋅⋅⋅O interactions (weak hydrogen‐bonding between anion oxygen and different CHs – predominantly CHs alpha to phosphorous). b) Packing diagram of [P_4441_][OAc] showing the CH⋅⋅⋅O interactions. c) Unit cell of [P_4441_][OAc] showing weak interactions (CH⋅⋅⋅O hydrogen‐bonding and van der Waals interactions between *n*‐butyl chains). d) Packing diagram (super cell) of [P_4441_][OAc] down the b‐axis (space filling representation). Alternating regions of weak CH⋅⋅⋅O hydrogen‐bonding and van der Waals interactions are apparent.

As mentioned earlier, it was possible to crystallise a pristine crystal from the melt for single‐crystal X‐ray structure determination. [P_4441_][OAc] crystallized in the triclinic space group P‐1 (No.2) with 4 crystallographic independent formula moieties in the asymmetric unit (a=13.4041(6) Å, b=13.9134(6) Å, c=21.0829(8) Å, *α*=73.704(2)°, *β*=87.204(2)°, *γ*=69.138(2)°, V=3520.8(3) Å^3^). Several *n*‐butyl substituents and one acetate anion are disordered. Due to the disorder, the structural parameters could not be discussed in detail. A model, not including the disorder, at the stage of the isotropic refinement of the non‐hydrogen atoms (except the anisotropically refined P‐atoms), with hydrogen atoms in calculated positions (‘riding’ model) is used for the calculation of powder diffraction patterns (Figure 12f) and the discussion of the structural features (R1=15.4 %). There are weak hydrogen‐bonds between the carboxylate O‐atoms and mainly *α* CH‐groups of the [P_4441_] alkyl chains, in the range of 2.20 to 2.60 Å (Figure 8a,b) as well as van der Waals interactions between the alkyl chains (Figure 8c,d). These bonding patterns are alternating, with clear regions of polar interactions *vs*. non‐polar interactions (Figure 8d) highlighting the amphiphilic nature of these materials. This is of course similar to the amphiphilic nature of cellulose, as demonstrated by both the native crystalline forms of cellulose I*β* ^[34]^ and I*α* ^[35]^ and the synthetic cellulose II.^[36]^

By contrast, the hydrogen‐bonds formed, from the crystal structures of King *et al*.^[11]^ and Parviainen *et al*.,^[10]^ using superbase‐derived carboxylate ionic liquids, range from 1.79‐1.98 Å (O⋅⋅⋅H) and 2.67‐2.78 Å (O⋅⋅⋅H−N). Thus, the strength of the hydrogen‐bonding between anion and cation for the superbase‐derived series is much stronger, however, the number of close contacts, through weak hydrogen‐bonding, in the [P_4441_][OAc] crystalline lattice is higher, approaching 5 close contacts per oxygen atom.

Calculation of the proton affinities for corresponding phosphonium ylide, DBN and [emim]‐carbene, according to a previous method by Parviainen *et al*.^[10]^ can highlight the contribution of the acidity of the cation towards this hydrogen‐bonding: proton affinities for the [P_4441_]‐ylide, [P_4444_]‐ylide, [emim]‐carbene (singlet) and DBN are 289.25, 294.16, 246.44 and 262.90 kcal/mol respectively. Clearly the *α* positions on the phosphonium salts are much less acidic than [emim]‐C2 and even more so [DBNH]‐NH, which accounts for the weaker hydrogen‐bonding interactions. However, as there is little transfer of charge, through hydrogen‐bonding and the fact that most of the formal positive charge on the phosphonium cation is located on the phosphorous atom, there is expected to be a very strong columbic interaction between the ion‐pairs. The phosphorous atom is also rather sterically shielded. This combined with the low cation acidity most likely make it difficult for the ion‐pairs to separate and the anion to act in a charge‐stabilizing manner, *e. g*. through hydrogen‐bonds to cellulose hydroxyls. In the context of our previous discussion about the requirement for ion‐pair separation to allow for cellulose dissolution, small but highly dipolar aprotic solvents seem appropriate for separation of the tetraalkylphosphonium acetate ion‐pairs yet maintaining basicity of the anion for interaction with cellulose hydroxyls. The requirement for a hydrophobic component for stabilization of cellulose is also implied, which may also require some degree of ion‐pair separation.

### Morphology and Crystallinity of the Regenerated Cellulose

**Figure 9:**
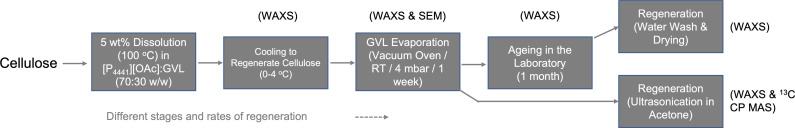
Analytical process scheme to determine regenerated cellulose crystallinity and morphology: Cellulose is first dissolved and regenerated by cooling, GVL is evaporated, and the cellulose is then fully regenerated either rapidly into acetone with ultrasound or slowly in water by washing away the ionic liquid.

**Figure 10:**
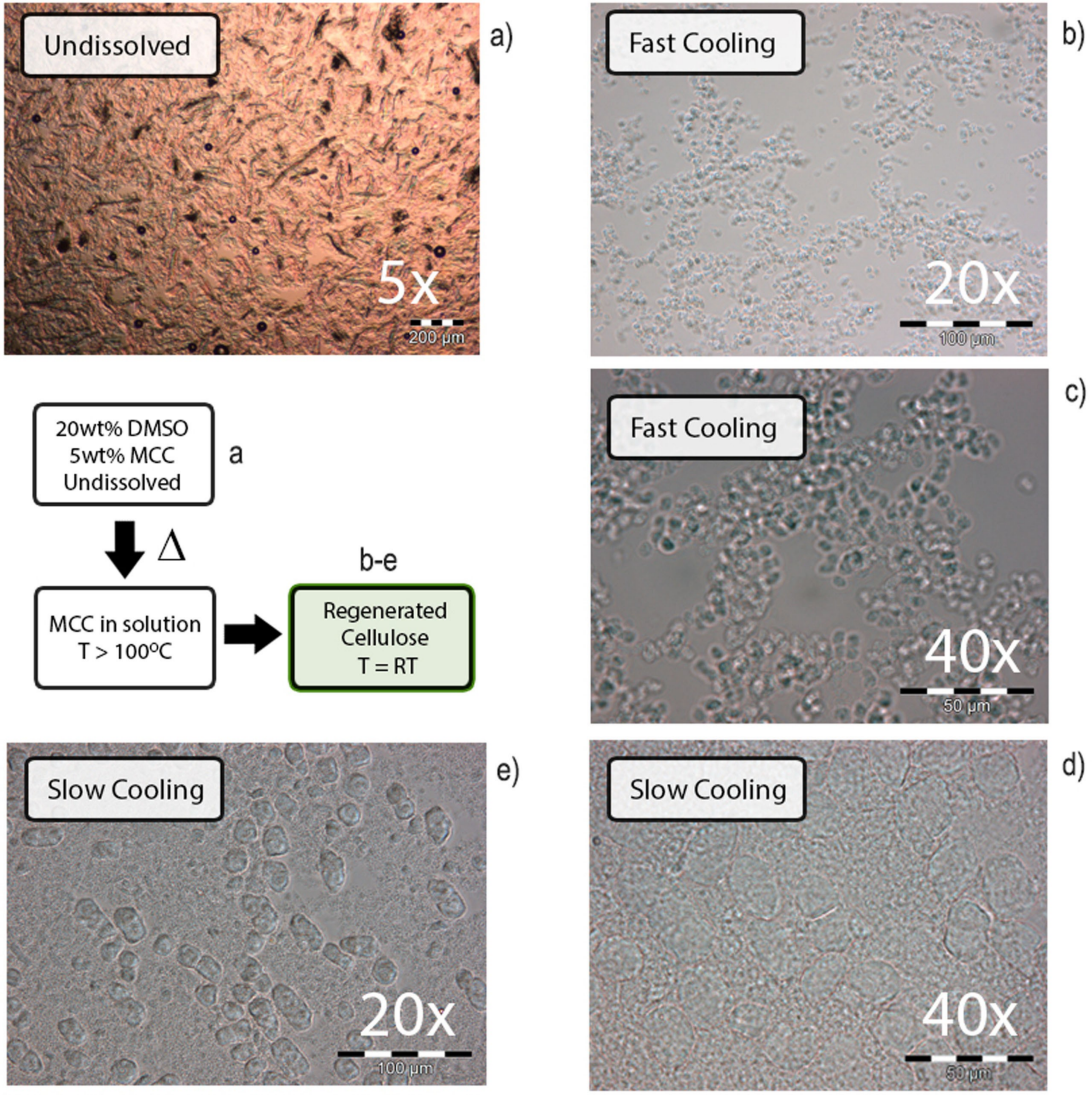
Variation in particle size upon regeneration of 5 wt% MCC from [P_4441_][OAc]:DMSO (80 : 20 w/w) at different cooling rates; slow cooling by letting sit on the bench and fast cooling by cooling in an ice bath.

Samples were regenerated at different cooling rates and with different water contents. Different methods were used to remove ionic liquid and co‐solvent, and finally to regenerate cellulose for morphology, and crystallinity determination (Figure 9), to better understand the mechanism of this phase‐behaviour. In more dilute MCC solutions (2‐6 wt %, in [P_4441_][OAc]:DMSO 80 : 20 w/w), roughly spherical micro‐scale structures are formed and can be observed *via* simple optical microscopy (Figure 10), upon cooling from the solution below the SGTT. These kinds of spherulites have been observed previously, for systems comprised of cellulose in: 1) *N*‐methylmorpholine‐*N*‐oxide (*N*MMO) hydrates,^[37]^ 2) the ionic liquid 1‐allyl‐3‐methylimidazolium chloride ([amim]Cl),^[38]^ 3) from aqueous solution by crystallization of cellooligomers, after enzymatic polymerization of *β*‐cellobiosyl fluoride,^[39]^ and 4) by regeneration from tetrabutylphosphonium acetate ([P_4444_][OAc]) electrolyte solutions.^[40]^ This last article focuses on a similar electrolyte system to the present article, which is more robust than the current system for regeneration of uniform particles.

Typically, in polymer solutions with an LCST or UCST, as the solvent becomes poor the polymer moves from a random coil to a collapsed globule conformation and, without stabilisation, the globules aggregate into larger particles. Here, the aggregation behaviour appears to be related to the cooling rate, similar to some synthetic polymer mixtures.[^41^, ^42^] In a 5 wt% MCC sample in [P_4441_][OAc]:DMSO (80 : 20 w/w), that was left to cool to room temperature over several hours, larger (with an average diameter of ca. 16 μm) structures are observed (which are initially more spherical and after some period, appear to breakup and become more oblique) in addition to what appears to be many smaller (ca. 1–2 μm) particles. For comparison, formation of multimolecular aggregates of polystyrene in cyclohexane, below the critical point, occurs within seconds.^[43]^ In a sample rapidly cooled in an ice bath, a more homogenous presentation of smaller scale (ca. 5.2 μm) spherical structures is observed (Figure 10). Clearly, the cooling rate and temperature influences the size and shape of the structures and the aggregation behaviour, which can be further exploited to produce spheres of different sizes. When we tried to isolate the micro particles, we quickly found that this was a challenge. It was not possible to filter the gel and when we added various co‐solvents to wash away the ‘residual ionic liquid’ the system simply gelled further. This indicated that the micro particles were only a small fraction of the cellulose in the mixture and that the bulk cellulose in the gel was likely in a slow state of regeneration, after the initial micro particle formation.

We also found it quite difficult to get consistent results upon repeating the experiments. Therefore, we were prompted to look at the influence of water as an impurity, upon the formation of the microparticles. It was found that on increasing the water content (1‐5 wt%) had dramatic effect on the particle size upon cooling (Supporting Information, S12). Increasing water content in the 5 wt% MCC [P_4441_][OAc]:DMSO (70 : 30 w/w) system tended to significantly increase the particle size. However, too much water results in a roughly opaque gel, with no microparticle formation. This complicates the reproducibility of the results, as these types of basic ILs have the tendency to rapidly absorb moisture from the atmosphere. Therefore, future studies into this topic need rigorous methodology for storing/transferring ILs and measurement of water contents.

**Figure 11:**
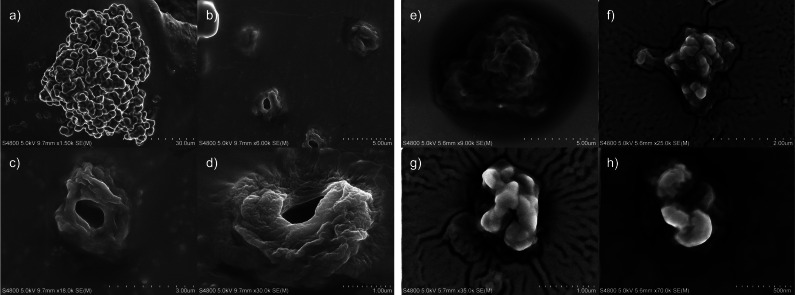
SEM analysis of cellulose microparticles after regeneration from [P_4441_][OAc]:GVL (a–d) and washing away of residual [P_4441_][OAc] using acetone and ultrasonication (e–f): a) microparticle cluster, b–d) separated microparticles retained on the support after flushing with nitrogen, e‐h) aggregated and fully regenerated microparticles of different sizes after washing away the [P_4441_][OAc].

To understand better the structuring of cellulose in the gel and upon full regeneration, the 5 wt % MCC in [P_4441_][OAc]:GVL (70 : 30 w/w) was dissolved and regenerated by rapid cooling (ice bath). The GVL was then evaporated away in a vacuum oven for SEM analysis. The particles showed visible clusters of microparticles (Figure 11a). These were found to contain a significant amount of [P_4441_][OAc] (at least on the surface). When the surface of the particles was illuminated by the electron beam the particle surface was observed to slowly melt. When the gel particles were blown away with pressurised nitrogen some particles remained on the carbon tape support, that seemed to be separated from the clusters (Figure 11b–d). These showed the micro particles that have a hollow centre and resembling the ‘globular’‐like particles observed in the optical microscopy (Figure 10).

**Figure 12:**
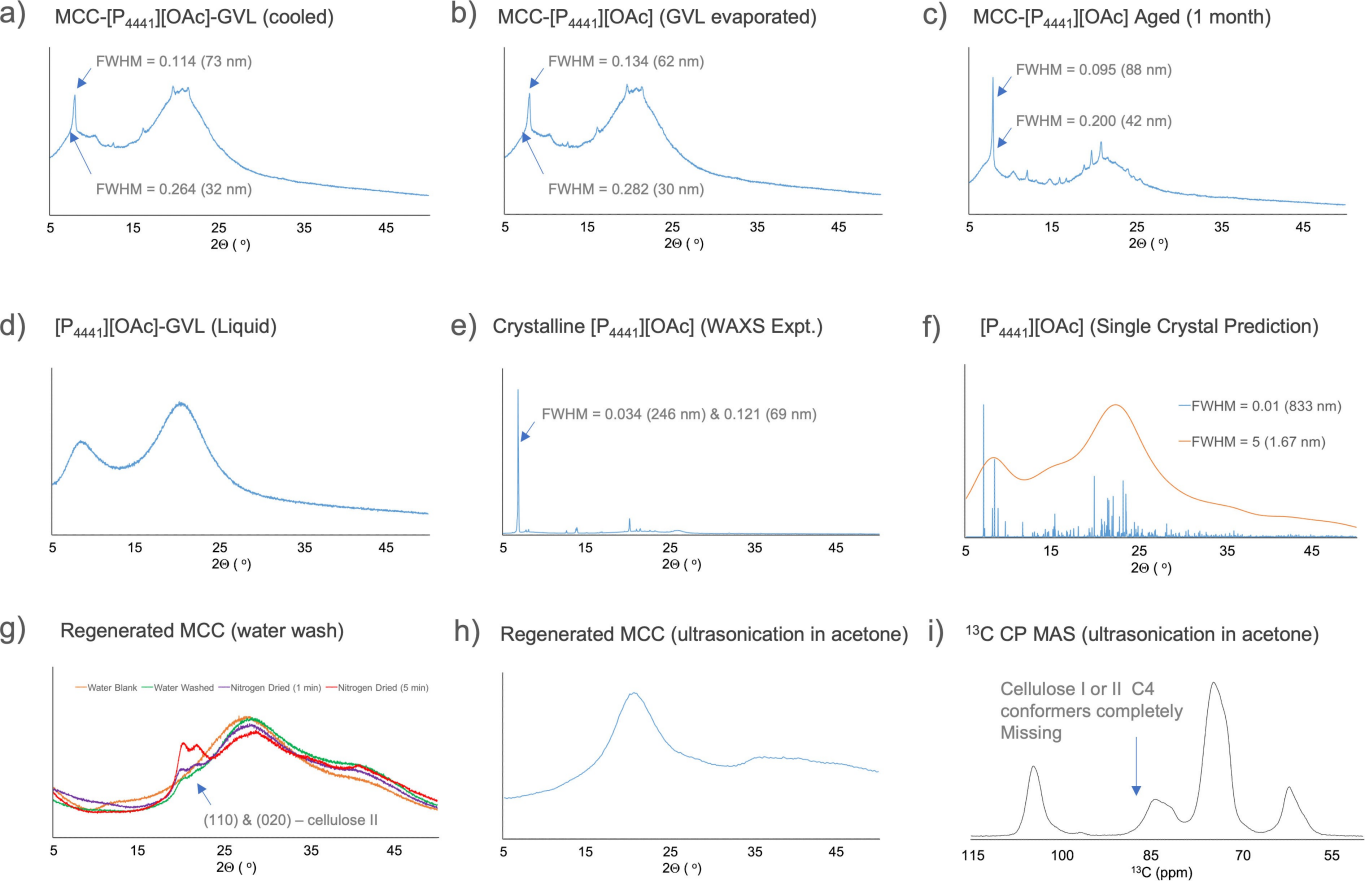
Cellulose crystallinity determination (through WAXS and ^13^C CP MAS NMR) for different samples after different stages of regeneration from [P_4441_][OAc]:GVL involving: a) initial cooling to regenerate microparticles, b) evaporation of GVL in a vacuum oven, c) ageing of the evaporated sample for 1 month, g) regeneration of cellulose only by washing away [P_4441_][OAc] with water and drying under nitrogen gas stream, h) and i) fast regeneration of the cellulose from [P_4441_][OAc] by ultrasonication in acetone.

To continue the regeneration and analysis, the cellulose‐[P_4441_][OAc] mixture (after GVL evaporation) was aged to observe further changes. Samples were then then completely regenerated to pure cellulose using two different methods: 1) slow regeneration into water by soaking over 2 weeks and evaporation of water under a nitrogen stream, 2) fast regeneration into acetone with ultra‐sonication. WAXS analysis was performed on all samples and the acetone‐regenerated sample was also analysed by ^13^C CP MAS NMR (Figure 12). For reference, WAXS analysis was also performed on the neat electrolyte and [P_4441_][OAc] which quickly crystallised from the melt (Figure 12). A defined crystal for single‐crystal x‐ray determination was also obtained from the melt, which had slowly crystallised in the fridge over a period of 4 months. From this, predicted X‐ray diffractograms were also calculated and used as reference (Figure 12). Rough crystallite sizes were determined for the crystalline [P_4441_][OAc] in the samples.

After the initial dissolution and regeneration by cooling (Figure 12a) there are no visible diffraction peaks corresponding to cellulose I or II.^[44]^ The broad dispersions centred at 8 and 22° corresponds to the background solvent (Figure 12d). There are however sharp peaks that do not correspond to the common Miller index planes for crystalline cellulose and are much larger in size (bimodal: at least 32 & 73 nm) than expected for regenerated cellulose crystallite dimensions. Similarly, when we simulate the diffraction pattern (FWHM=0.01, using Mercury) from the single‐crystal x‐ray diffraction crystallographic information file (cif) we also see sharp peaks at ∼8°, corresponding mainly to the (010) plane. What is also interesting is that by simulating the diffraction pattern for a reduced crystallite size (FWHM=5), broad dispersions – similar to the background OES – are observed (Figure 12d). One could conclude that, as the cellulose regenerates, there is co‐crystallisation of [P_4441_][OAc] containing structurally similar motifs to those found in either of the two crystalline [P_4441_][OAc] samples (single crystal and polycrystalline, see above). This is further exemplified by analysis of the 1‐month aged sample (Figure 12c), where we can observe enhancement of the signal intensity corresponding to the large crystalline planes at ∼8°. This is concomitant with a decrease in the broad signals, corresponding to background solvent. However, for the aged sample, the 8° is shifted to a slightly lower 2θ value. This likely does not result from changes in sample height calibration, as the sharper signals at higher 2θ values are also different. Rather, it seems that there has been a conversion to a different, but similar, allomorph. This may be aided by uptake of moisture from the atmosphere, during the 1 month of ageing. Nevertheless, this crystalline phase has increased, and the crystallite sizes are also significantly larger. Song *et al*.[^45^, ^46^] have published a similar spherulite regeneration from cellulose dissolved in [amim]Cl. There also observe similar sharp diffraction peaks for the co‐cellulose‐IL phase and propose the formation of an extensive crystalline phase (with large crystallite sizes corresponding the sharp diffraction signals) where [amim]Cl is incorporated into the unit cell. While such large polymer crystallite sizes are not typically observed for cellulose, they do occur.^[47]^

To investigate this further, the non‐dissolving cellulose‐[P_4441_][OAc] (GVL evaporated) samples were fully regenerated to cellulose using the two methods described above. The first, where the sample is simply soaked in water for 2 weeks, on the x‐ray stage holder, formed a gel where the OES slowly diffused out into the water. This was analysed (Figure 12g) showing mainly the water background and two small but broad peaks at 21 and 23°. No sharp signals, that may correspond to preservation of existing chain orientations (producing large plane size) upon slow diffusion of OES out of the crystalline phases, were observed. The sample was then dried under a compressed nitrogen gas stream for initially 1 min and then 5 min. Between and after the drying steps the water background, in the diffractograms (Figure 12g), was observed to decrease and the peaks at 21 and 23°, which are characteristic of the (110) and (020) planes in cellulose II,^[47]^ and cellulose II hydrate,^[48]^ increased. This increase is partly related to increase in concentration of the cellulose as water is evaporated from the gel. However, it is clear from this that the washing with water allows for eventual regeneration cellulose II, of considerably smaller crystallite size, typical of fast regeneration of cellulose‐IL solutions into water. Ultrasonication in acetone was applied, for comparison with the aqueous regeneration method. WAXS analysis (Figure 12h) shows no presence of cellulose I or II. The diffractogram is characteristic of completely amorphous cellulose. Furthermore, this is also confirmed by ^13^C CP MAS NMR, where the C4 peaks corresponding to cellulose I or II (centred at 88 ppm) are completely missing (Figure 12i). If we look at the SEM analysis for this acetone‐regenerated sample (Figure 11e–h), there is a broad dispersion of sizes of particles from the micro‐scale and even into the nano‐scale. It seems as if any microparticles that have formed have collapsed or fragmented and material that was in the gel‐state has fully regenerated. Using this method, a broad dispersion of particle sizes of amorphous high surface‐area cellulose is accessible. Further studies are required to confirm the co‐crystallisation of cellulose and IL, cellulose‐IL crystallisation, or some combination of both, during particle formation.

## Conclusions

Thermo‐responsive regeneration of cellulose has been demonstrated using mixtures of the tetraalkylphosphonium acetate ionic liquid [P_4441_][OAc] with either DMSO or GVL, with the pure ILs not capable of dissolving cellulose over non‐destructive temperatures. The thermodynamics of the sol‐gel transition are similar to those of UCST‐type phase‐transitions. The SGTT is shown to be dependent on solvent composition, trace water content and cellulose concentration, potentially offering the possibility of production of tuneable micro dispersions of cellulose spherical particles. Recovery of such phases, in the solid‐state seems complicated but tantalising, due to the potential to control particle sizes from the nano‐mm range. The regeneration seems to be dependent on our ability to tune the solvent quality by varying co‐solvent composition but also choice of co‐solvent, with more dipolar aprotic co‐solvents leading to better quality solutions.

## Conflict of interest

The authors declare no conflict of interest.

## Supporting information

As a service to our authors and readers, this journal provides supporting information supplied by the authors. Such materials are peer reviewed and may be re‐organized for online delivery, but are not copy‐edited or typeset. Technical support issues arising from supporting information (other than missing files) should be addressed to the authors.

Supporting Information
