# Supplementary material for: Thermo‐Reversible Cellulose Micro Phase‐Separation in Mixtures of Methyltributylphosphonium Acetate and γ‐Valerolactone or DMSO
Source: Chemphyschem. 2022 Feb 25;23(7):e202100635. doi: 10.1002/cphc.202100635 (PMC9303658; doi:10.1002/cphc.202100635)
Supplement: Supplementary file 1 — Supporting Information [file CPHC-23-0-s001.pdf]

ChemPhysChem

Supporting Information

Thermo-Reversible Cellulose Micro Phase-Separation in Mixtures of Methyltributylphosphonium Acetate and γ-Valerolactone or DMSO

Ashley J. Holding, Jingwen Xia, Michael Hummel, Harry Zwiers, Matti Leskinen, Daniel Rico del Cerro, Sami Hietala, Martin Nieger, Marianna Kemell, Jussi K. J. Helminen, Vladimir Aseyev, Heikki Tenhu, Ilkka Kilpeläinen,* and Alistair W. T. King*

Supporting Information

Table of Contents

S1. Materials 2

S2. Synthesis of Ionic Liquids 2

[P_{4441}][OAc] 2

[DBNH][OAc] 4

S3. Crystal Structure Determination & Parameters for [P_{4441}][OAc] 4

S4. Regeneration Experiments 8

Preparation of Regenerated Micro Particles in [P_{4441}][OAc]:GVL for WAXS 8

Preparation of Regenerated Micro Particles in [P_{4441}][OAc] for WAXS 8

Complete Regeneration of Micro Particles from Water for WAXS 9

Complete Regeneration of Micro Particles from Acetone for WAXS and ^{13}C CP MAS NMR 9

Regeneration of Micro Particles for SEM Analysis 9

S5. WAXS Experimental 9

S6. Optical Microscopy 10

S7. Scanning Electron Microscopy 10

S8. ^{13}C CP MAS NMR 10

S9. DSC 10

S10. Rheology 11

Dynamic Modulii, Heating 13

Storage Modulii, Heating and Cooling 15

Complex Viscosity, Heating and Cooling 16

S11. Kamlet-Taft Parametisation 18

S12. Influence of water content on particle size 20

S13. Transmittance measurement by UV-spectrophotometer 23

S1. Materials

MCC (Microcrystalline Cellulose DP_n: 87.03, DP_w: 356.94, PDI: 4.14) was purchased from Sigma Aldrich Ltd, Enocell Pre-Hydrolysis Kraft Pulp was provided by Storä Enso (DP_n: 468, DP_w: 1560, PDI: 3.33). Tributylmethylphosphonium methyl carbonate solution (80% in Methanol) was purchased from Iolitec GmBH. Glacial acetic acid (>98.5wt%), was purchased from Sigma Aldrich Ltd. Reichardt's Dye (2,6-Diphenyl-4-(2,4,6-triphenyl-1-pyridinio)phenolate), *N,N*-diethylnitroaniline and 4-nitroaniline dyes for Kamlet-Taft measurements were purchased from Sigma Aldrich Ltd. 1-ethyl-3-imdazolium acetate ([emim][OAc], >98%) was purchased from Iolitec GmBH.

S2. Synthesis of Ionic Liquids

$[P_{4441}]$[OAc]

Glacial acetic acid (165.13 g, 2.73 mol) was added, dropwise to tributylmethylphosphonium methyl carbonate, 80 wt% in methanol 799.19 g (2.73 mol) evolving carbon dioxide. The reaction mixture was stirred at room temperature for ca. 1 day after which methanol was evaporated (in batches) at 60°C under reduced pressure. The product, tributylmethylphosphonium methyl acetate, was dried under high vacuum at 80°C, in roughly equal batches, for around 24 hours. Yield: 96.2% M.P = 40.02 ^{o}C (Determined by DSC).

NMR spectra were recorded using a Varian 600 (600MHz ^{1}H Freq.) spectrometer. Pre-relaxation delay times of 30 s were used, to ensure complete relaxation of phosphonium alpha CH_2/CH_3 (2.3-2.5 ppm) and acetate-CH_3 (1.9-2.0 ppm) protons for integration. For ^{13}C and HSQC spectra, a 3s delay time was used.

^{13}C NMR (600 MHz, DMSO-D_6) δ 172.13 (s, $CH_3\underline{C}O_2^-$), 25.61 (s, $\underline{C}H_3CO_2^-$), 23.30 (s, -$\underline{C}H_2$-CH_3), 22.61 (s, -$\underline{C}H_2$-), 18.59 (d, J=49.02 Hz, P-$\underline{C}$H2), 13.13 (s, -CH_3), 2.91 (d, J=51.06 Hz, P-$\underline{C}H_3$).

^{1}H NMR (600 MHz, DMSO-D_6) δ 2.26 (m, 6H, P-C$\underline{H_2}$-), 1.87 (d, 3H, J=14.6 Hz P-C$\underline{H_3}$), 1.54 (m, 3H, C$\underline{H_3}$$CO_2^-$), 1.45 (sex, 6H, J=7.12 Hz -C$\underline{H_2}$-), 1.38 (m, 6H, -C$\underline{H_2}$-CH_3), 0.89 (t, 9H, J=7.46 Hz -C$\underline{H_3}$).

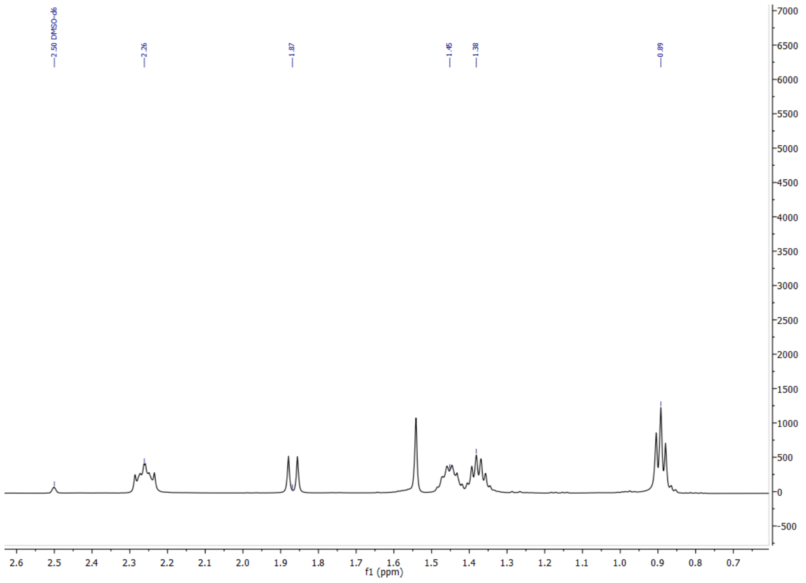

Figure S1: ^{1}H Spectrum of $[P_{4441}]$[OAc] in DMSO-d_6.

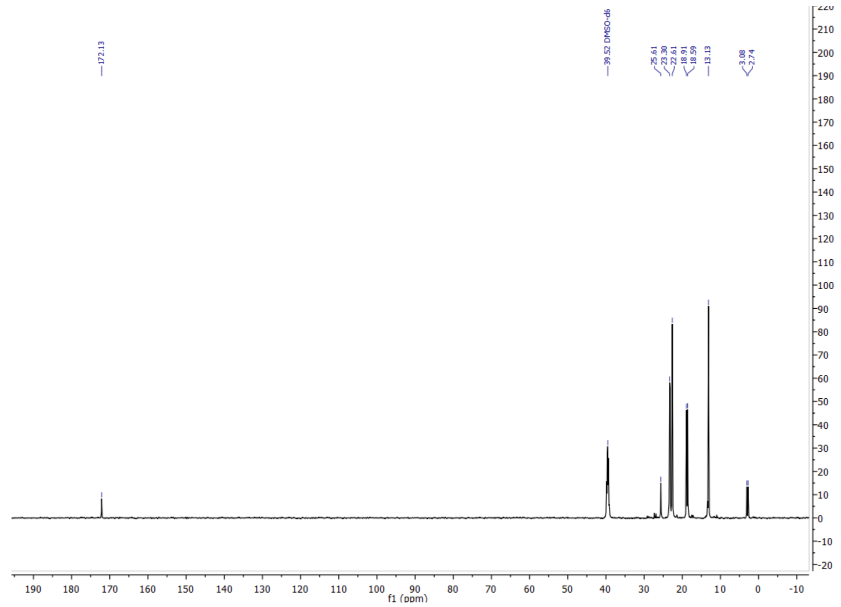

Figure S2: ^{13}C Spectrum of $[P_{4441}][OAc]$ in DMSO-d_6.

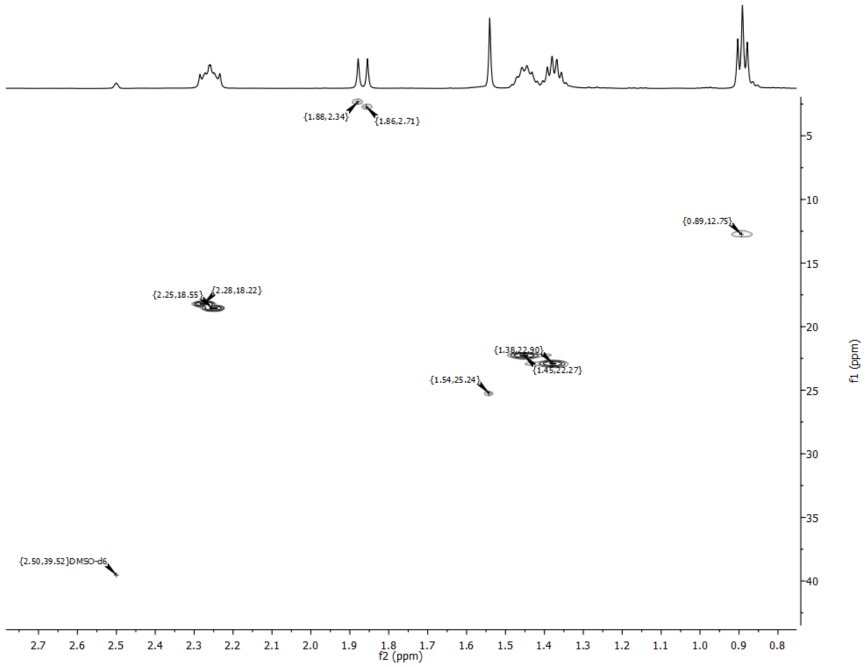

Figure S3: HSQC Spectrum of $[P_{4441}][OAc]$ in DMSO-d_6.

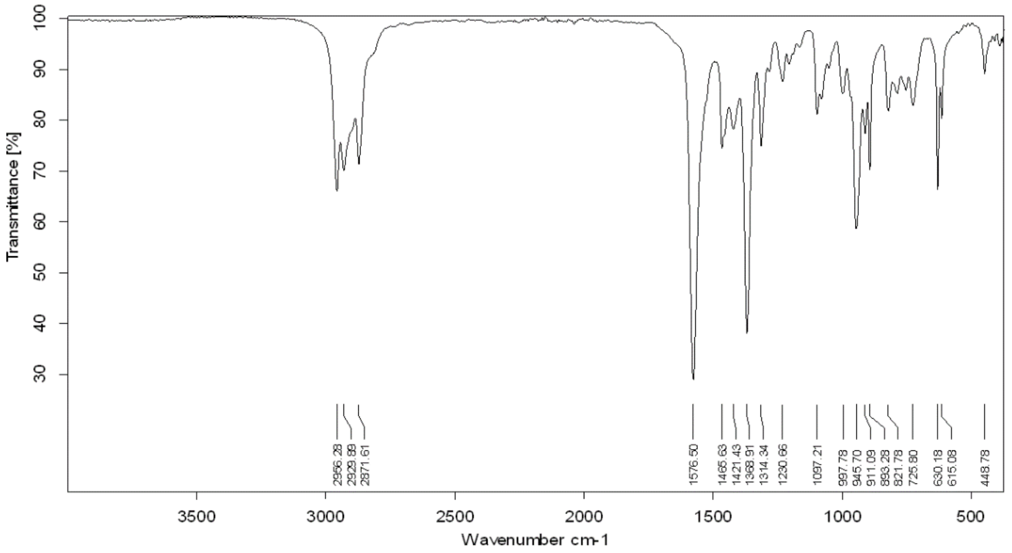

Figure S4: FT-IR Spectrum of pure [P_{4441}][OAc].

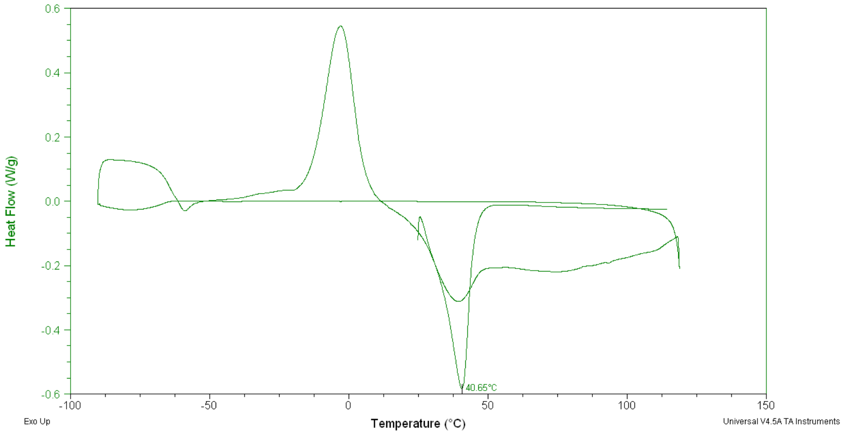

Figure S5: DSC Thermogram of pure [P_{4441}][OAc].

[DBNH][OAc]

The [DBNH][OAc] sample was synthesised previously and is described in the previously published procedure.[10] (see main text)

S3. Crystal Structure Determination & Parameters for [P_{4441}][OAc]

The single-crystal x-ray structure for [P_{4441}][OAc] was determined after the compound crystallised from the ionic liquid melt. [P_{4441}][OAc] crystallized in the triclinic space group P-1 (No.2) with 4 crystallographic independent formula moieties in the asymmetric unit (a = 13.4041(6) Å, b = 13.9134(6) Å, C = 21.0829(8) Å, α = 73.704(2)°, β = 87.204(2)°, γ = 69.138(2)°, V = 3520.8(3) Å3). Several n-butyl substituents and one acetate

anion are disordered. Due to the disorder, the structural parameters could not be discussed in detail. A model, not including the disordered, at the stage of the isotropic refinement of the non-hydrogen atoms (except the anisotropically refined P-atoms) with hydrogen atoms in calculated positions (ridong model) is used for the calculation of the powder diffraction pattern and the discussion of the structural features (R1 = 15.4%).

There are weak hydrogen bonds between the carboxylate O-atoms and CH-groups of the alkyl chains of [P_{4441}] in the range of 2.20 to 2.60 Å (see Fig. S6, Fig. S7) als well as van der Waals interactions between the alkyl chains (see Fig. S8-S10).

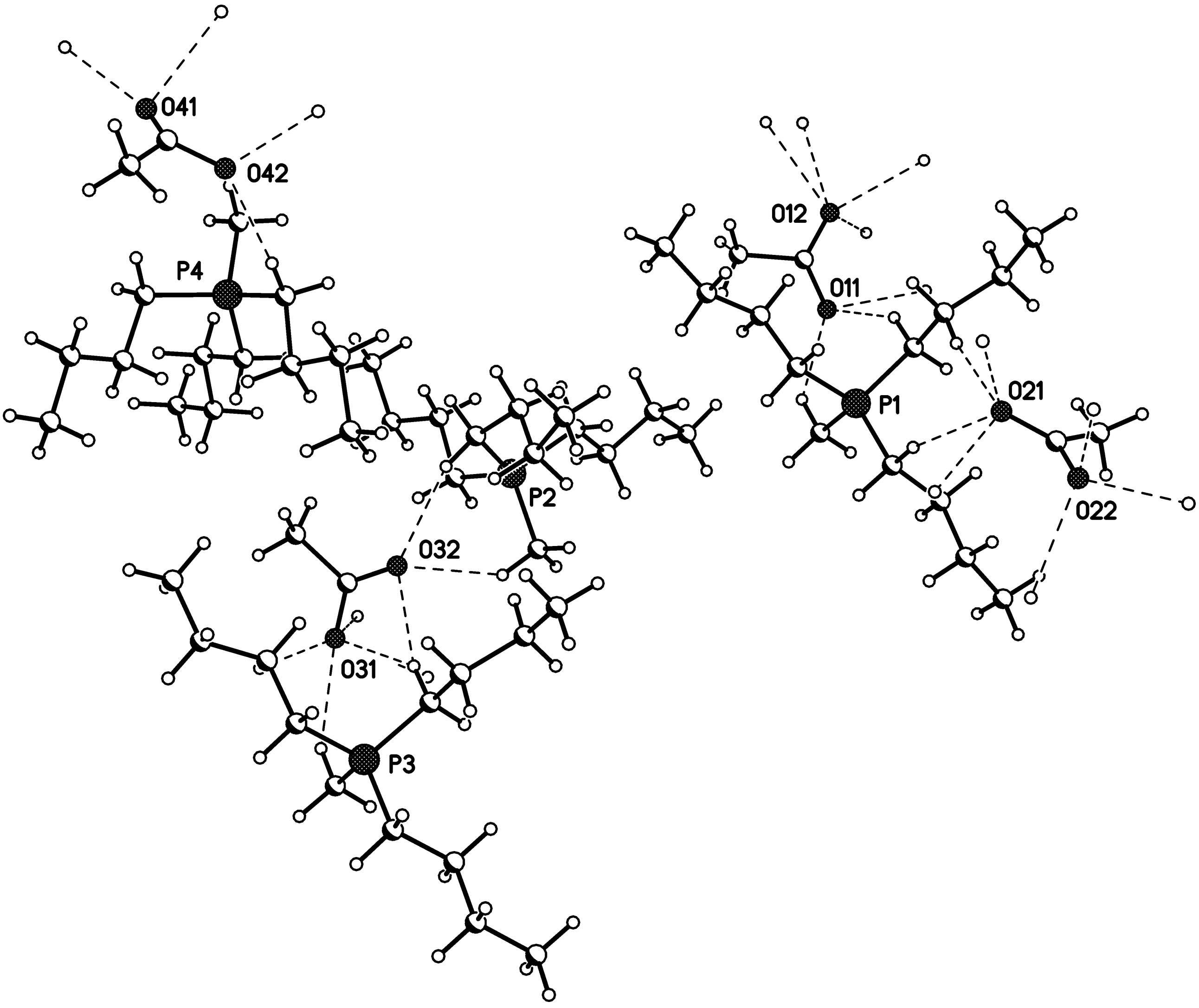

Figure S6. 4 crystallographic independent formula moieties of [P_{4441}][OAc] in the asymmetric unit showing the weak CH…O interaction.

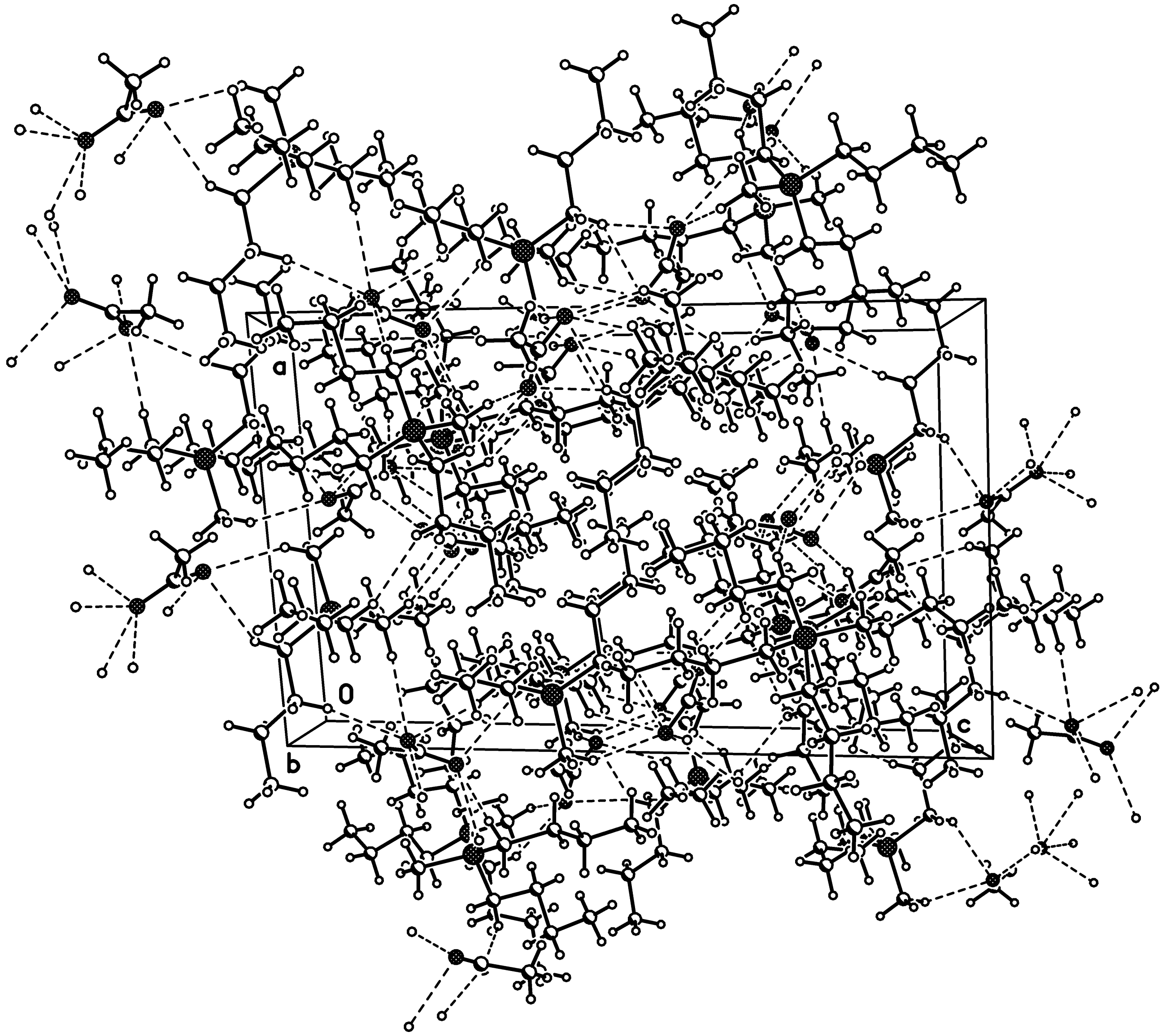

Figure S7. Packing diagram of [P_{4441}][OAc] showing the weak hydrogen-bonds.

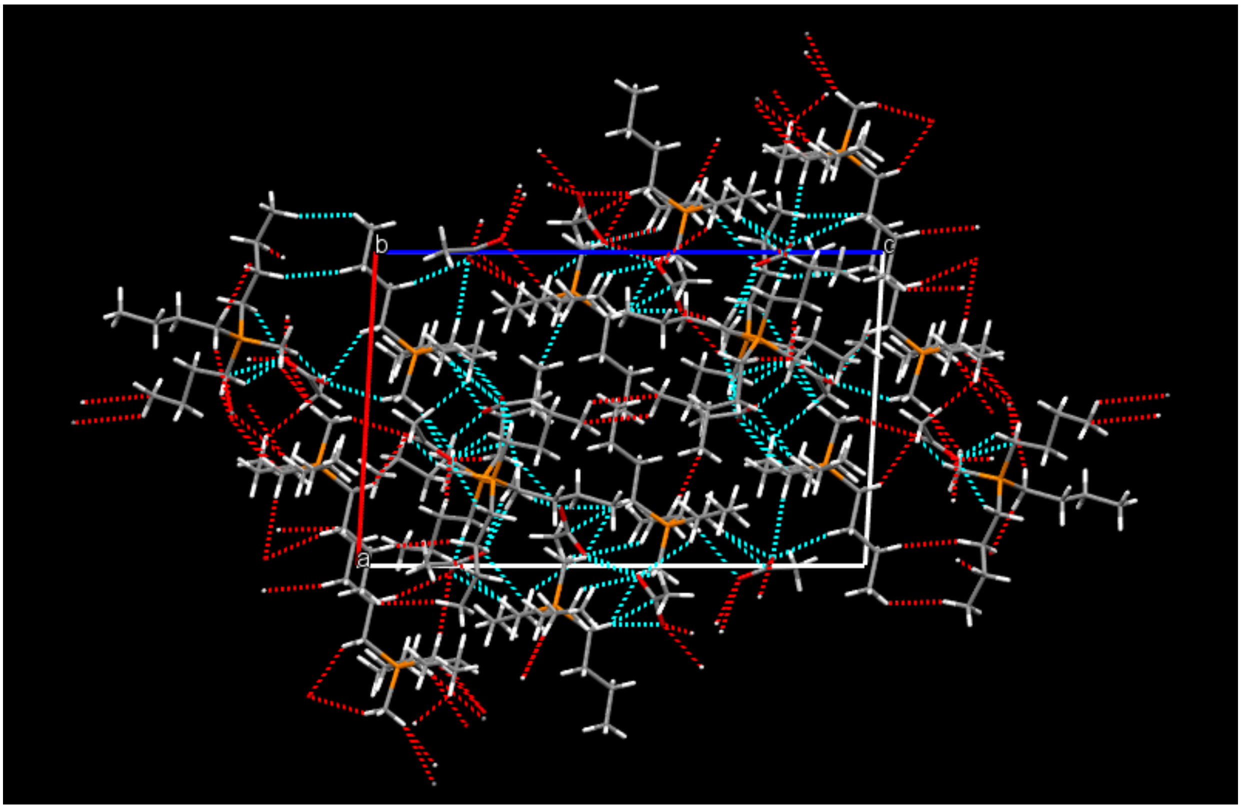

Figure S8. Unit cell of [P_{4441}][OAc] showing weak interaction (CH…O and von der Waals interaction).

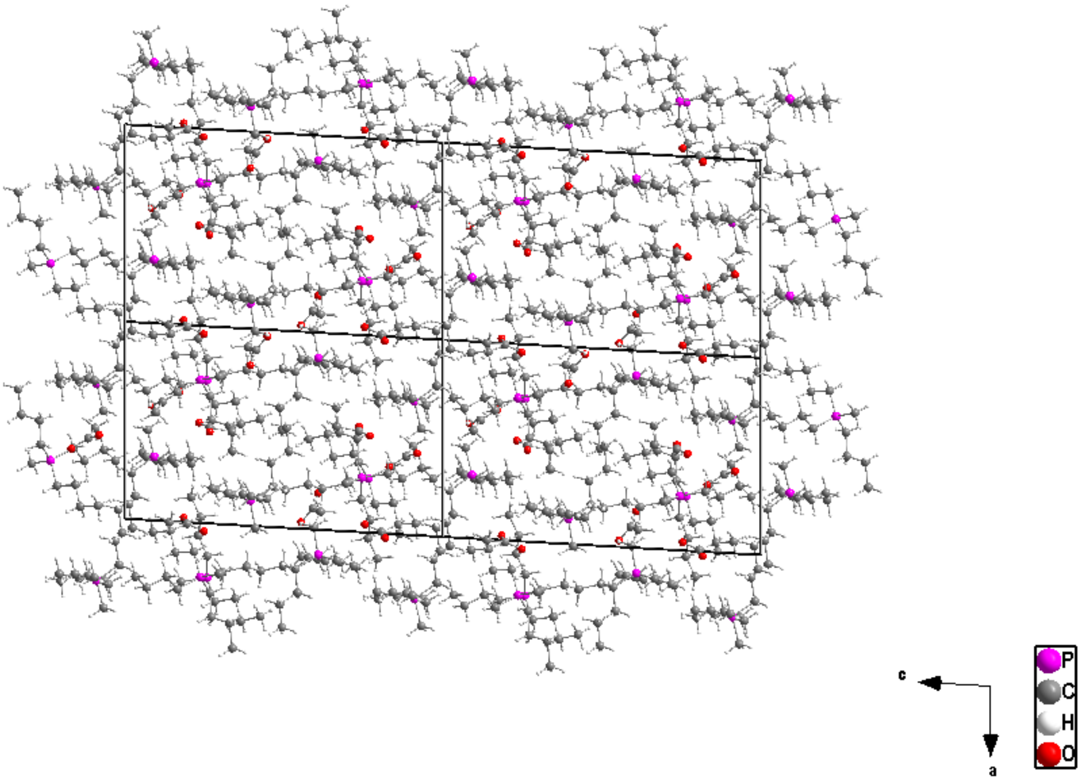

Figure S9. Packing diagram (super cell) of [P_{4441}][OAc] down the b-axis (ball and stick)

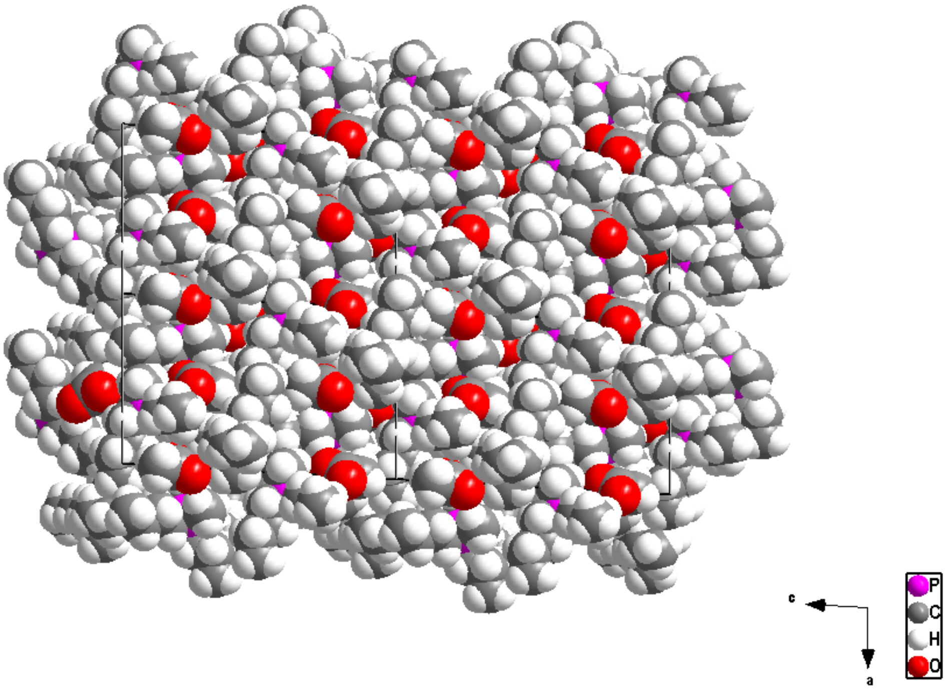

Figure S10. Packing diagram (super cell) of [$_{P4441}$][OAc] down the b-axis (ball and stick)

S4. Regeneration Experiments

Several samples were prepared using different cooling conditions. All show the same phase-transition phenomenon and microparticle formation:

Preparation of Regenerated Micro Particles in [P_{4441}][OAc]:GVL for WAXS

The dissolution step was performed as above in a sealed vial to yield a 5 wt % solution of MCC in [P_{4441}][OAc]:GVL (70:30 w/w). The sample was then cooled in, an ice water bath for 30 min, to rapidly regenerate the particles. This formed a viscous opaque yellow liquid. Subsequently, the sample was stored in the refrigerator (4 °C) for 72 h, after which the sample was then left to sit at room temperature for a few hrs, prior to WAXS analysis.

Preparation of Regenerated Micro Particles in [P_{4441}][OAc] for WAXS

The dissolution step was performed as above in a sealed vial to yield a 5 wt % solution of MCC in [P_{4441}][OAc]:GVL (70:30 w/w). The sample was then poured onto a petri dish and was placed in a vacuum oven (<5 mbar) at RT for 72 hr to evaporate the GVL. This formed a thick opaque yellow paste. The sample was stored at room temperature before WAXS analysis using the Rigaku equipment. The sample, as applied to the WAXS sample holder was then aged in the laboratory atmosphere at RT for ~ 1 month. A new WAXS diffractogram for this 'aged' sample was then recorded

Complete Regeneration of Micro Particles from Water for WAXS

The aged sample, prepared above, was left to soak in a water bath for 2 weeks. The sample was then removed from the water bath and a WAXS diffractogram measured before the sample dried. The sample was then dried using a pressurised nitrogen stream for 1 min and 5 min. WAXS analysis was performed after each stage.

Complete Regeneration of Micro Particles from Acetone for WAXS and ^{13}C CP MAS NMR

The dissolution step was performed as above in a sealed vial to yield a 5 wt % solution of MCC in $[P_{4441}]$[OAc]:GVL (70:30 w/w). The sample was stored in the refrigerator (4 °C) for 2 weeks, after which the sample was placed in a vacuum oven (<5 mbar) for 7 d to evaporate the GVL. This formed a thick opaque yellow paste. Acetone was (200 mg of paste in 40 ml of acetone) added and the sample was sonicated, using a Hielscher UP100H Ultrasonic Processor (tip sonicator), to aid in the removal of the ionic liquid from the cellulose particles. Centrifugation (4000 rpm, 10 min) was used to remove the solid particles and the procedure was repeated. The solid material was air-dried to yield a white powder. The sample was stored at room temperature before WAXS and ^{13}C CP-MAS analysis.

Regeneration of Micro Particles for SEM Analysis

The dissolution step was performed as above in a sealed vial to yield a 5 wt % solution of MCC in $[P_{4441}]$[OAc]:GVL (70:30 w/w). The sample was cooled in ice for 5 min to rapidly regenerate the particles. The sample was then stored in the refrigerator (4 °C) for 24 h, after which it was placed in a vacuum oven (< 5 mbar) for 48 hr to remove the GVL. This formed a thick opaque yellow paste. A small portion of this was used for SEM analysis. The paste was applied to carbon tape on the SEM support analysis area. SEM analysis of this material did not yield results as when the electron beam was applied to different regions the samples quickly melted. This was attributed to melting of the ionic liquid. The sample was then flushed with nitrogen gas to remove the surface layers of the sample. When the majority of the gel particles were blown away most particles remained on the carbon tape support in clusters, while some were on the support as individual particles.

To analyse the fully regenerated particles, free from ionic liquid, the vacuum dried sample (above) was treated with acetone (10 mg of paste in 40 ml of acetone) and ultrasonicated, using a Hielscher UP100H Ultrasonic Processor (ultrasonic probe), to aid in the removal of the ionic liquid from the cellulose particles. Centrifugation (4000 rpm, 10 min) was used to remove the solid particles. The pellet material was again sonicated in acetone (40 ml) and a small volume of this was then cast onto a silicon wafer and evaporated for SEM analysis.

S5. WAXS Experimental

A Rigaku SmartLab diffractometer was used for measuring the symmetric reflection x-ray diffractograms of the samples containing microparticles; either in electrolyte or the ionic liquid with GVL evaporated or regenerated in water and water evaporated with nitrogen. The samples were placed in a pre-calibrated 20 mm * 20 mm * 0.5 mm (depth) glass sample holder and the surface smoothed using a straight razor-blade. The aqueous regenerated and nitrogen-dried sample was dried under a stream of compressed nitrogen for the specified time. An x-ray beam of Cu K-alpha using multilayer monochromator was used. The data was

collected using a two-dimensional HyPix-3000 single photon counting detector at a distance of 300 mm at a speed of 15 degrees / min. A 2θ range between 5° and 50° was measured.

The dry acetone (ultrasound) regenerated particles were pressed into a pellet (50 mg) using an FT-IR KBr press. They were placed on a height-calibrated IR stage. The diffractograms were collected using a PANalytical X'Pert Pro MPD system, using Bragg-Brentano (reflectance) geometry. The diffracted intensity of Cu Kα radiation (λ = 1.54Å, under a condition of 45 kV and 40 mA) was measured in a 2θ range between 5° and 50°.

S6. Optical Microscopy

Olympus BX 51 optical microscope was used in conjunction with the software AnalySIS, from Olympus Soft, which was used to collect and process the resulting images and measure the size of the particles. A rough estimate of the particle size was made based upon the length of the horizontal diameter of a number of particles, accounting for the diffraction corona. A polarising cross filter was used to analyse birefringence of the cellulose crystals before and after dissolution and regeneration.

S7. Scanning Electron Microscopy

The SEM images were acquired with a Hitachi S-4800 field emission scanning electron microscope. The samples were coated with 5-10 nm of Au-Pd alloy prior to imaging.

S8. ^{13}C CP MAS NMR

Solid state ^{13}C CPMAS NMR spectra were measured using Bruker Avance III 500 spectrometer with magnetic flux density of 11.7 T, using a double resonance CPMAS probe-head. Samples were packed into 4 mm ZrO2 rotors and plugged with KEL-F endcaps and spun at spinning frequency of 10 kHz. The length of the contact time for cross-polarization was 1 ms and a variable amplitude cross-polarization ramped from 70% to a maximum amplitude during contact time was used. During the acquisition period the protons were decoupled using SPINAL-64 decoupling and the length of the acquisition was 27 ms. Scans were collected with a 3 s relaxation delay. The spectrum was referenced externally via adamantane by setting the low field resonance at 38.48 ppm.

S9. DSC

A *TA Instruments* DSC Q200 differential scanning calorimeter was used for preliminary DSC investigations into the thermal behaviour of the ionic liquid, molecular solvent and cellulose solutions, based on [P_{4441}][OAc]/DMSO. Hermetically sealed aluminium Tzero pans were used to contain ca. 10mg of sample. After optimising the method, including the temperature range, and heating and cooling rates, a Heat/Cool/Heat cycle was employed, heating from 20°C to 120 °C (at 5.00 °C/min), cooling to -50°C (at 5.00 °C/min), and heating to 120°C (at 5.00 °C/min).

S10. Rheology

Oscillatory shear rheology of all solutions was measured on an Anton Paar MCR 300 rheometer with a plate and plate geometry (25 mm plate diameter, 1 mm gap distance). The viscoelastic domain was determined by performing a dynamic strain sweep test and a strain of 1%, which fell well within the linear viscoelastic regime, was chosen for the frequency sweep measurements. Each sample was subjected to dynamic temperature sweep measurement at an angular frequency of 1 s^{-1} and strain of 1%. The temperature range was adjusted depending on the [P_{4441}][OAc]/GVL/cellulose system and cooling/heating rates of 2–4 °C/min were chosen.

The sol-gel transition temperatures were taken as the intersection between the dynamic moduli – storage and loss modulus, upon heating (Example, Figure S 11). An additional measure of the sol-gel transition temperature was the inflection point of the complex viscosity curve upon heating. This additional measure of the SGTT is given below (Figure S12)

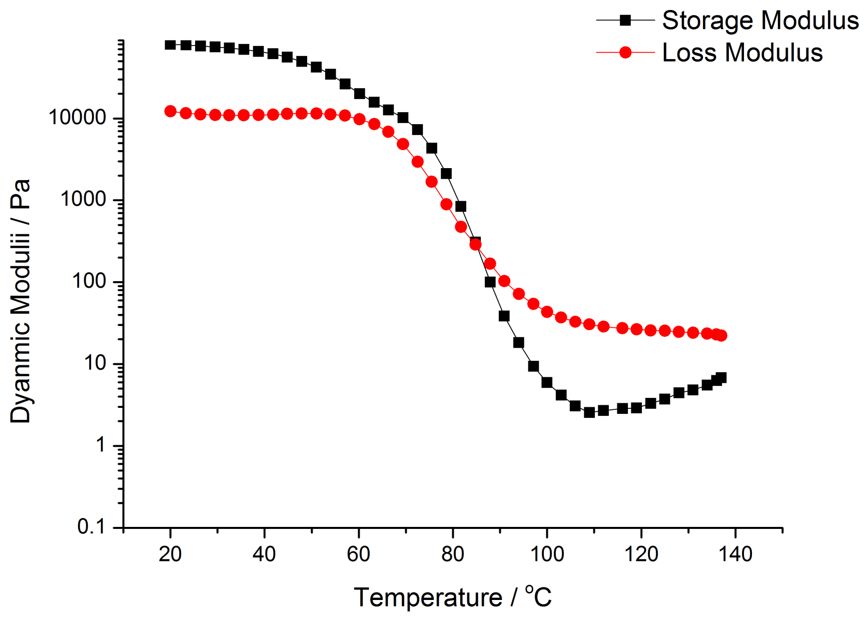

Figure S11: Example of Sol-Gel Transition temperature, with crossover of Dynamic Modulii

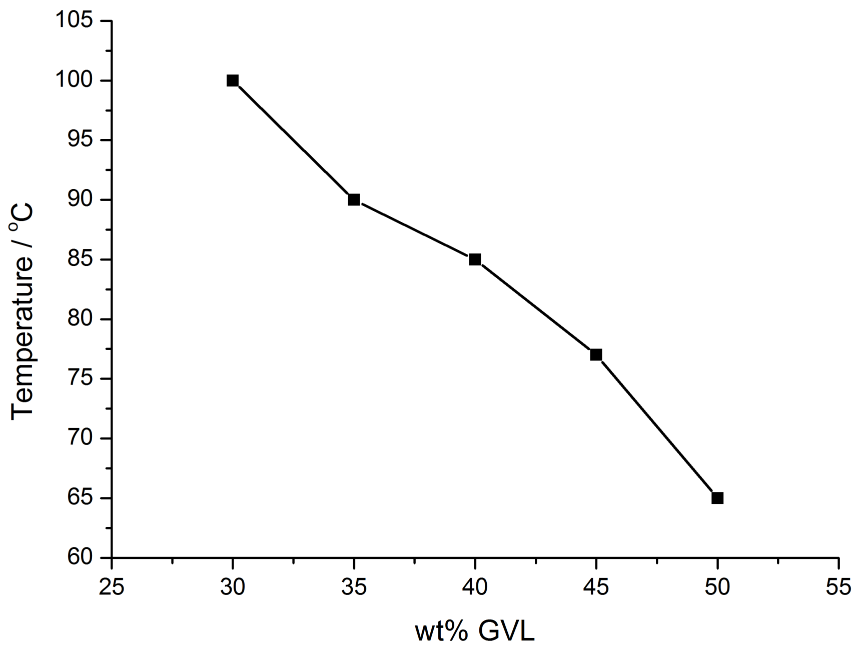

Figure S12: Alternative Measure of Sol-Gel Transition: Inflection Point of Complex Viscosity Curves

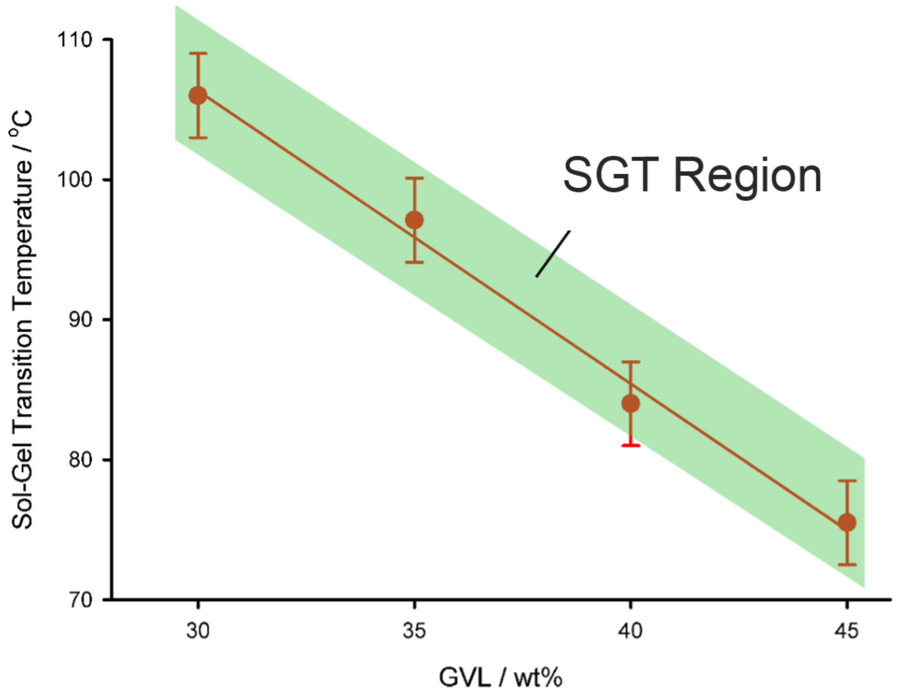

Figure S13: Sol-Gel Transition Temperature versus GVL wt%

Dynamic Modulii, Heating

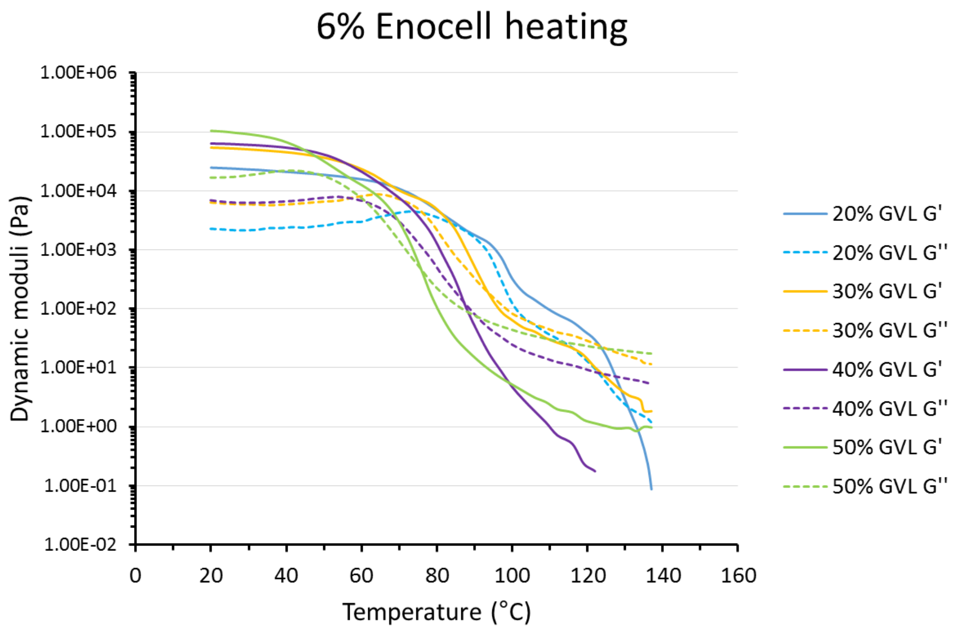

Figure S14: Dynamic Moduli, Heating, 6wt% Enocell and varying GVL wt% range

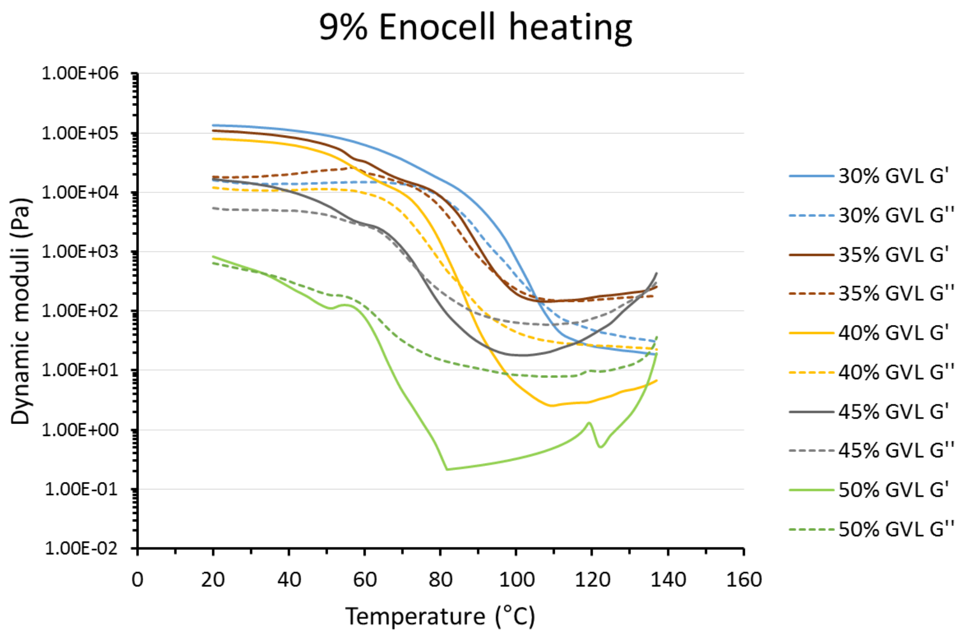

Figure S15: Dynamic Moduli, Heating, 9wt% Enocell and varying GVL wt% range

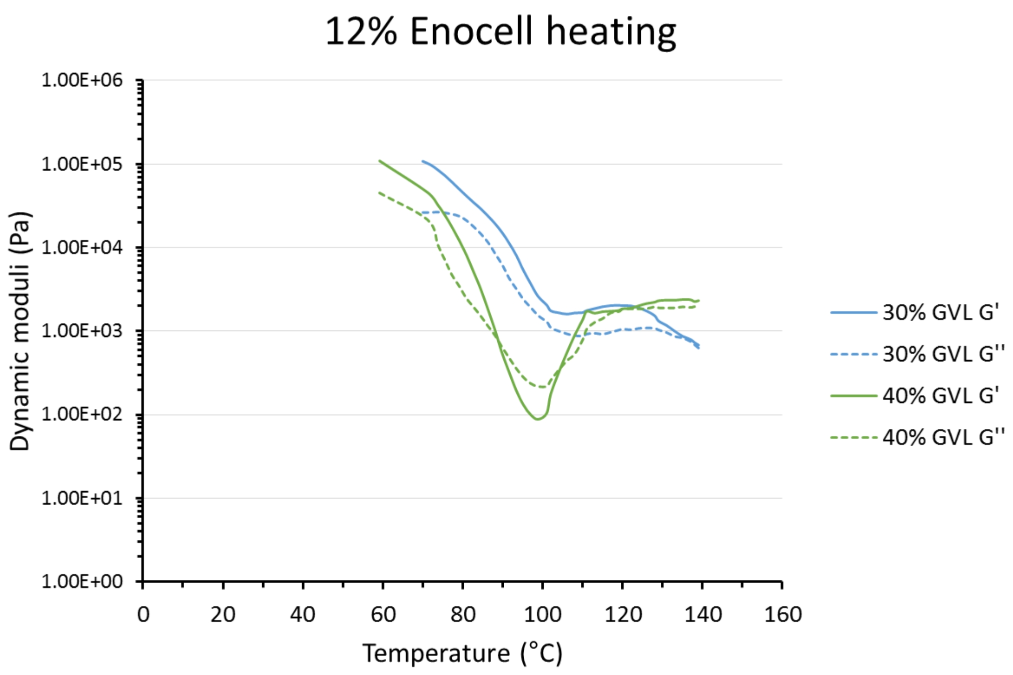

Figure S16: Dynamic Moduli, Heating, 12wt% Enocell and varying GVL wt% range

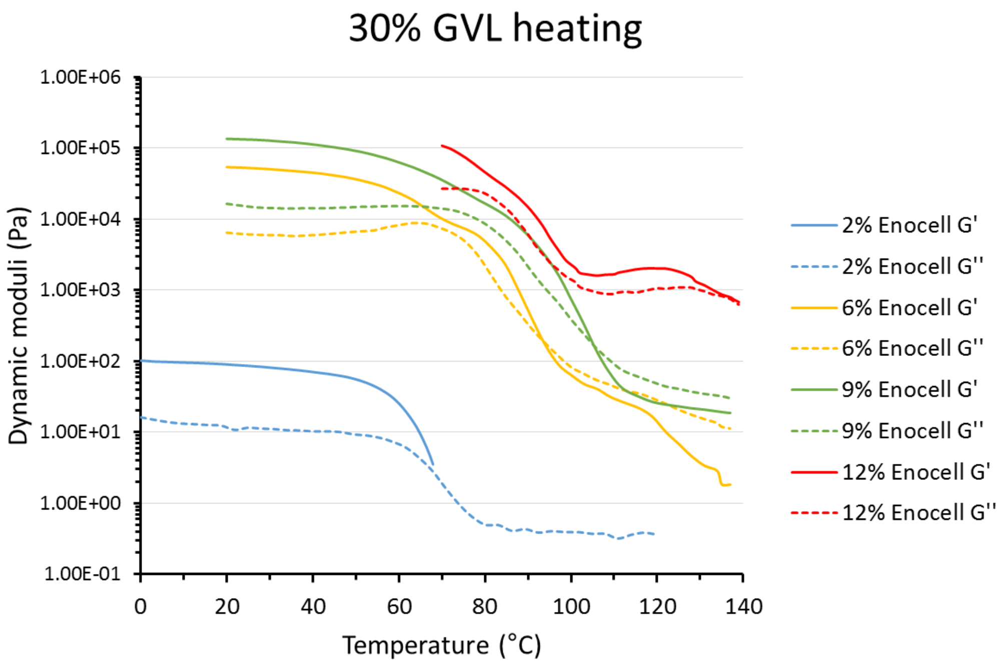

Figure S17: Dynamic Moduli, Heating, 30 wt% GVL with varying range of Enocell wt%

Storage Modulii, Heating and Cooling

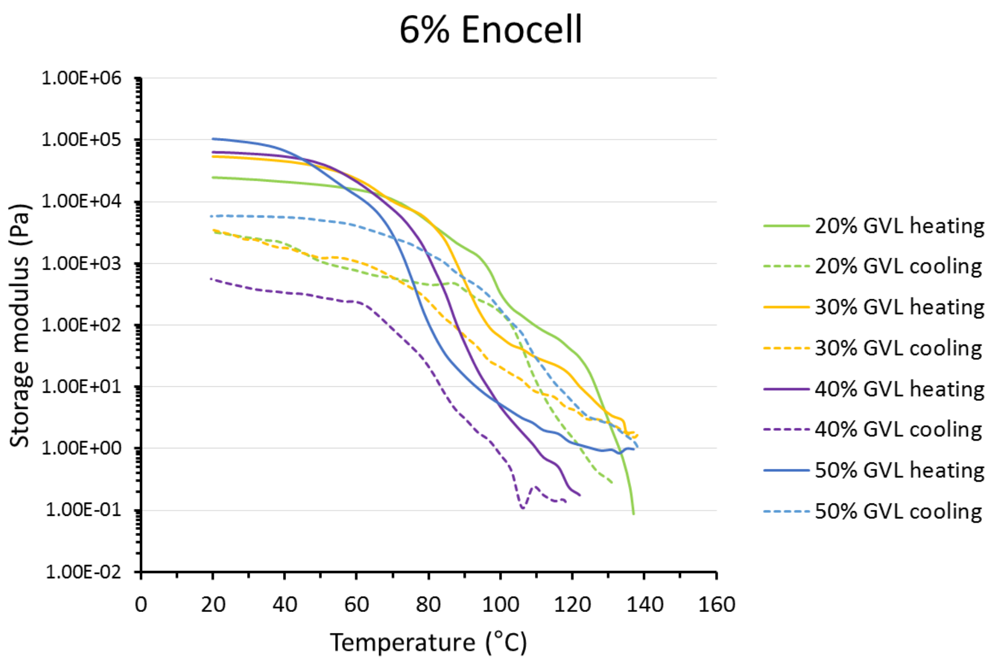

Figure S18: Storage Modulus, Heating and Cooling, 6wt% Enocell in with varying range of GVL wt%

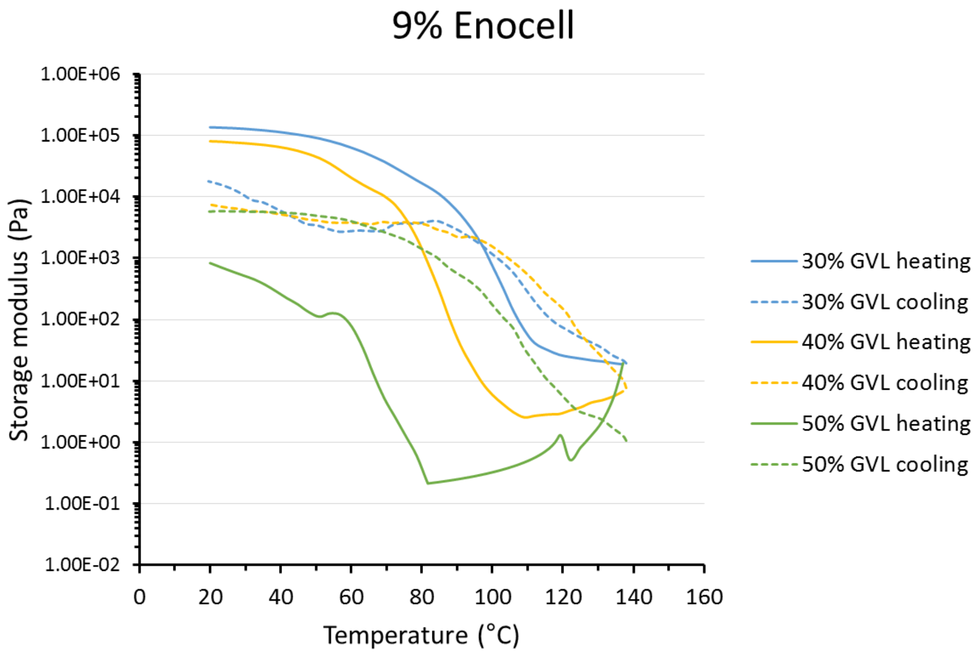

Figure S19: Storage Modulus, Heating and Cooling, 9wt% Enocell in with varying range of GVL wt%

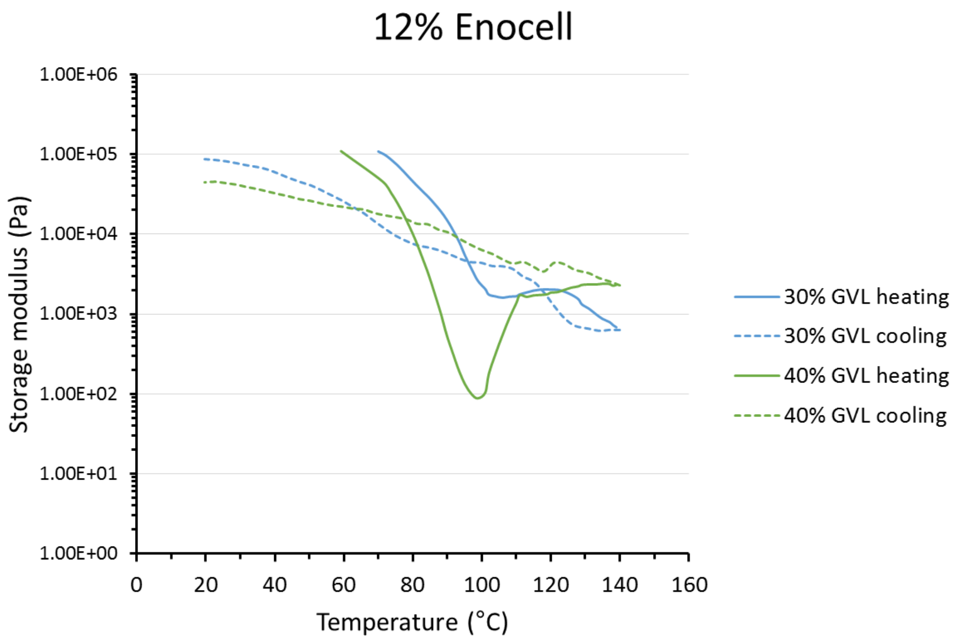

Figure S20: Storage Modulus, Heating and Cooling, 12 wt% Enocell in with varying range of GVL wt%

Complex Viscosity, Heating and Cooling

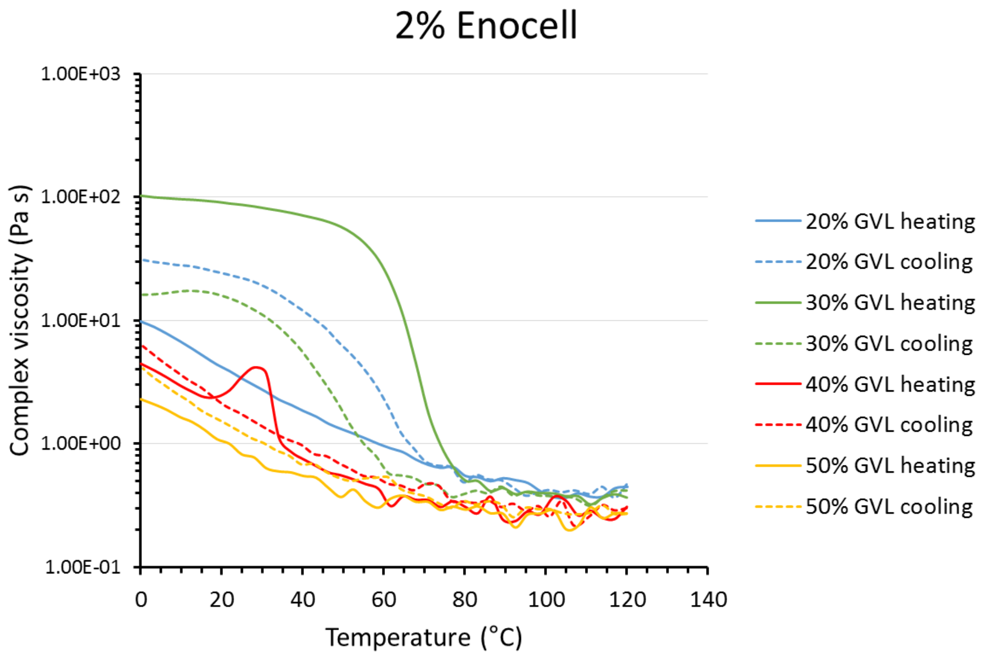

Figure S21: Complex Viscosity, Heating and Cooling, 2wt% Enocell in with varying range of GVL wt%

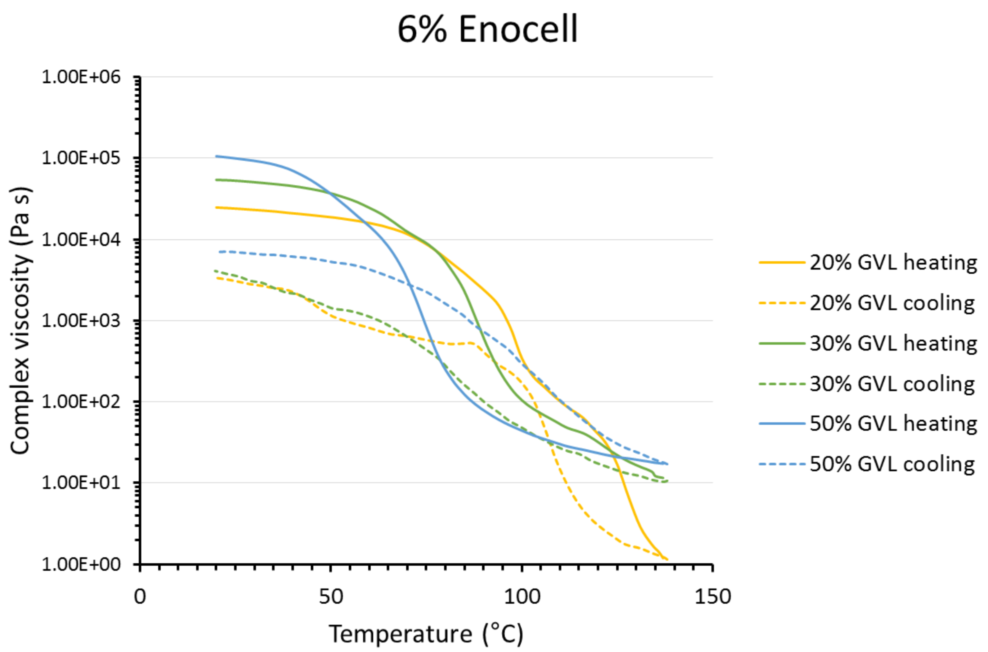

Figure S22: Complex Viscosity, Heating and Cooling, 6wt% Enocell in with varying range of GVL wt%

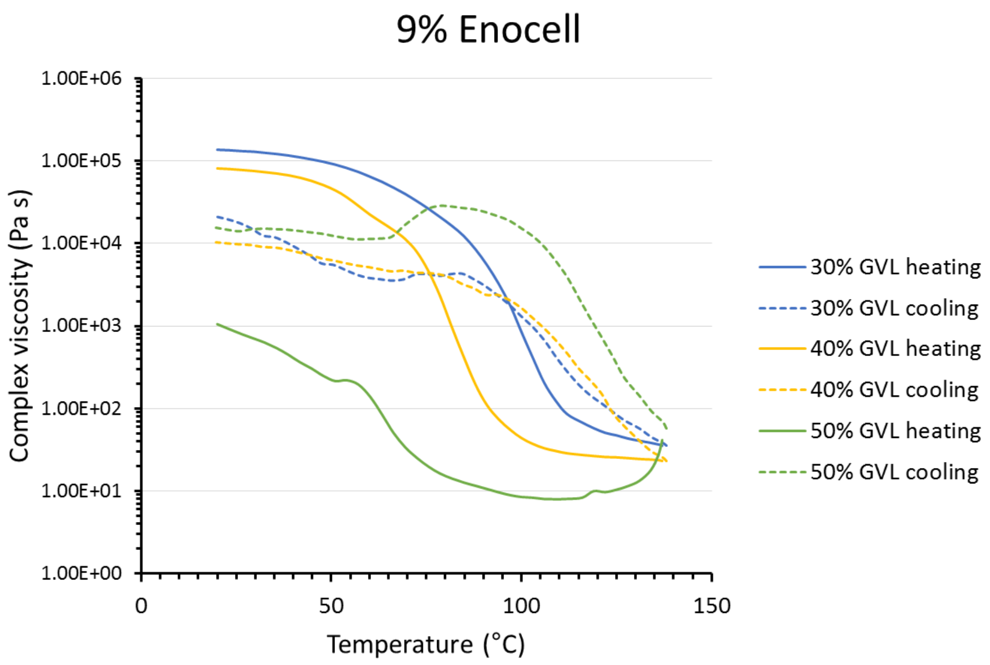

Figure S23: Complex Viscosity, Heating and Cooling, 9wt% Enocell in with varying range of GVL wt%

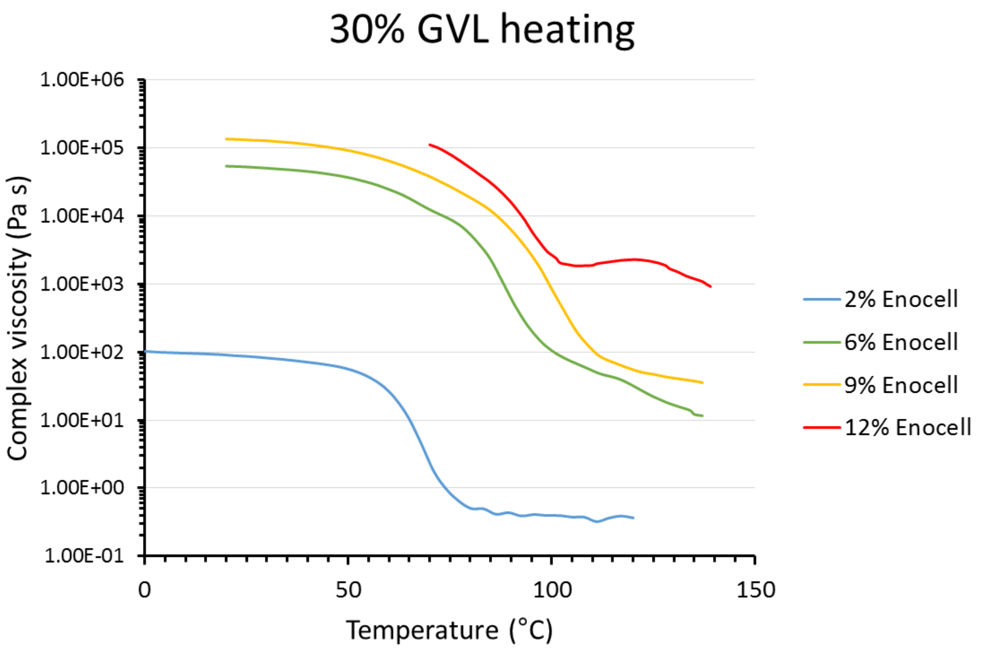

Figure S24: Complex Viscosity, Heating and Cooling, 30 wt% GVL with varying range of Enocell wt%

S11. Kamlet-Taft Parametisation

Three standard solutions of three dyes, Reichardt's Dye (2,6-Diphenyl-4-(2,4,6-triphenyl-1-pyridinio)phenolate), *N,N*-diethylnitroaniline and 4-nitroaniline were made in acetone.

Dye solutions (ca 10-20 µl) were introduced into vials and evaporated, before introducing the ionic liquid or electrolytes into the vials, and mixing, using heat if necessary, to form homogenous solutions. The solutions were transferred to 1mm quartz cuvettes. For each sample and dye combination, frequency-sweeping UV spectra were collected from λ 300-800 nm. A polynomial function was fitted to the curve maxima at λ_{max} enabling a more precise λ_{max} to be calculated. The Kamlet-Taft parameters were calculated as outlined in our previous publication.[4] (see main text)

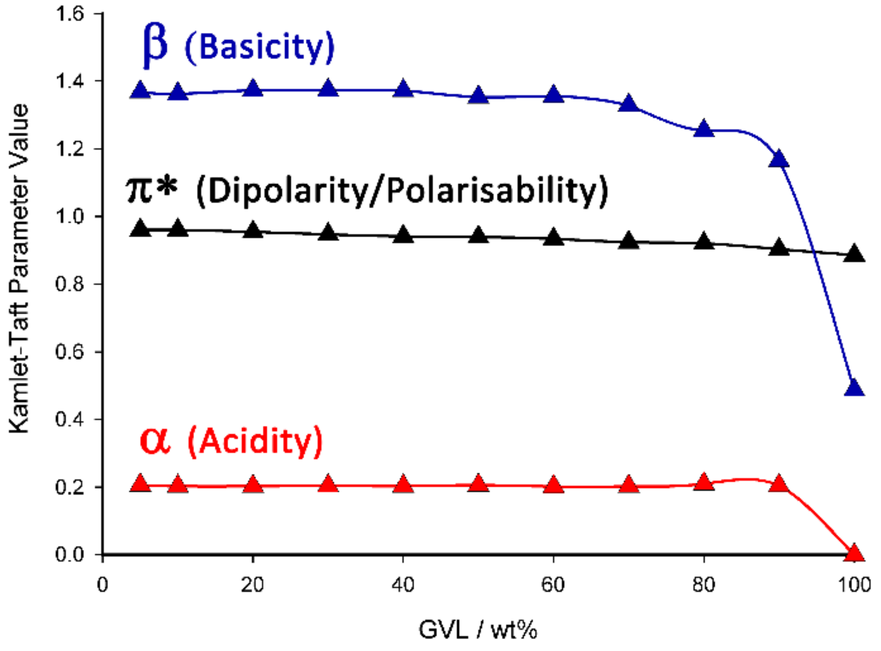

Figure S25: KT parameters, GVL and [P_{4441}][OAc]

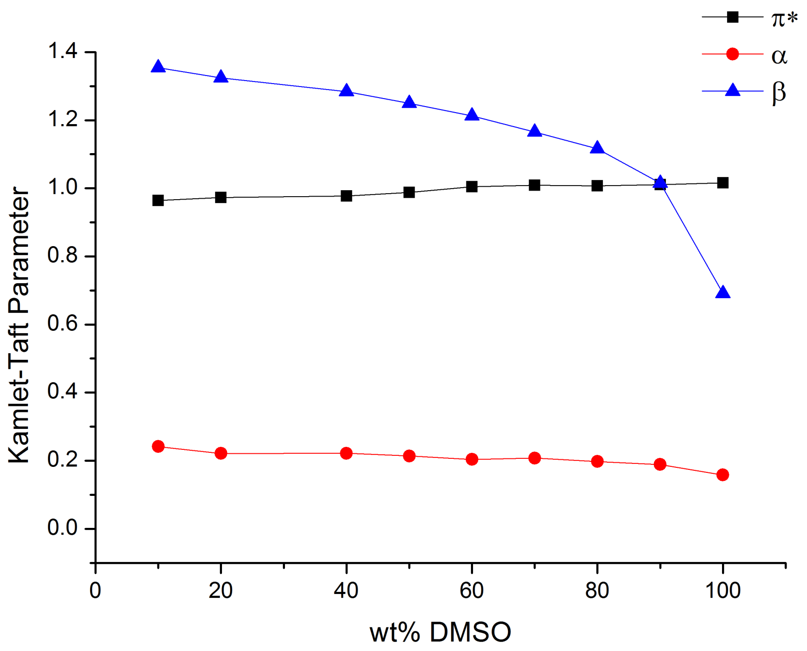

Figure S26: KT parameters, DMSO and [P_{4441}][OAc]

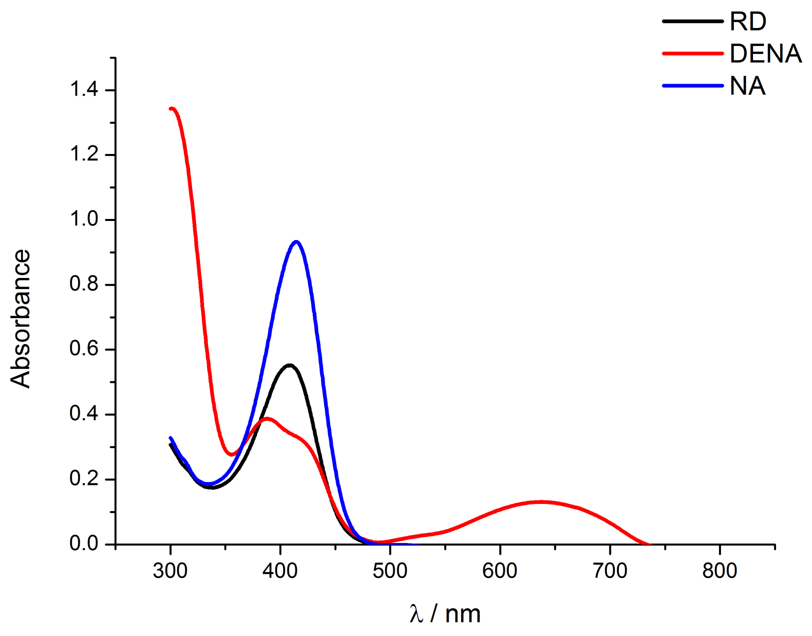

Figure S27: Example UV spectra of dyes in solution, 40wt% GVL and [P_{4441}][OAc]. RD: Reichardt's Dye, DENA: N,N-diethylnitroaniline, NA: 4-nitroaniline

S12. Influence of water content on particle size

Trace water was demonstrated to be a factor that influences the particle forming rate and particle size. Eight different regenerated cellulose solutions were prepared (5wt% MCC dissolved in electrolyte: [P_{4441}][OAc]:DMSO 70:30 w/w): containing 0 wt%, 0.1 wt%, 0.5 wt%, 1.0 wt%, 2.0 wt%, 5.0 wt% & 10 wt% water. The cellulose solutions were pre-heated at 60°C for 45 minutes with stirring to pre-disperse the cellulose. These were then transferred to a 120°C oil bath and heated for 5 minutes. The solutions were then allowed to cool naturally at room temperature and allowed to sit overnight for regeneration.

Two different procedures were applied to test if the water addition time has effect on the regenerated cellulose particles. *a)* 1 wt% water was added in the electrolyte before adding cellulose and pre-dispersing, *b)* 1 wt% water was added in the electrolyte after dissolving cellulose at 120°C – water addition was at ~100 °C during cooling. The following Figure 28 shows obvious differences between these two methods. Larger and more numerous particles are regenerated if the water is added before dispersing cellulose. According to these results, procedure *a)* was applied in the following water wt% experiments.

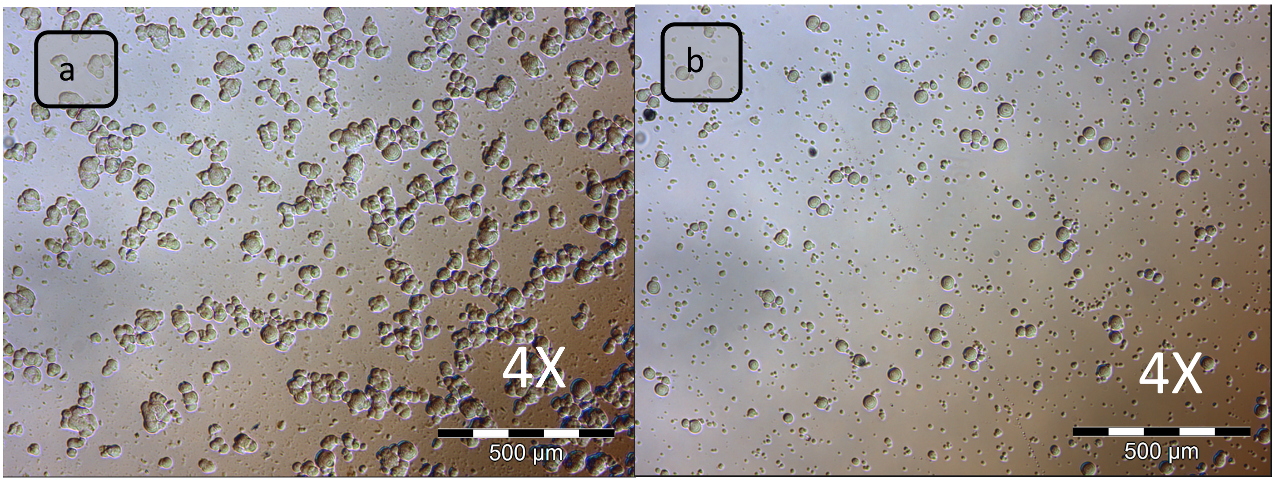

Figure S28. Regenerated cellulose solution (with 1 wt% water) under microscope; a) water added before pre-dispersing; b) water added after cellulose dissolution

The regenerated cellulose solutions with and without 1 wt% water were checked after 3 hours (Figure S29). The cellulose solution with 1 wt% water was opaque while the one without water was still clear, indicating that the microparticle regeneration rate was much faster than without water. In addition, within the same heating time, the colour of the cellulose solution without water turned darker. The sample with water did not darken.

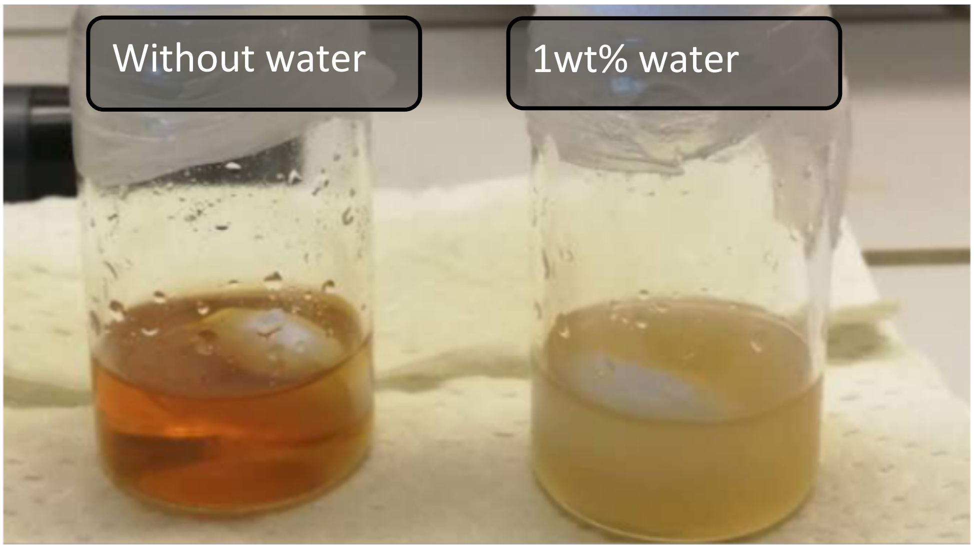

Figure S29. Appearance of solutions after resting for 3 hours in room temperature: 5 wt% MCC in [P_{4441}][OAc]: DMSO(70:30 w/w) with 1 wt% water and without water

After studying the full water concentration range, the cellulose cannot be dissolved if the water concentration was too high (between 5-10 wt%). 5 wt% water samples require longer to dissolve (8 minutes). After resting overnight at room temperature, opaque regenerated cellulose solutions were obtained. The following Figure 30 shows the appearance of the regenerated cellulose particles with gradient of water content (0 wt%, 0.1 wt%, 0.5 wt%, 1 wt%, 2 wt%, 5 wt%) taken using an optical microscope. The increasing in water content gives rise to larger regenerated cellulose particles. In addition, the particle shapes become irregular when the concentration of water is over 1 wt%. The difference in size between cellulose solutions with 0.1 wt% water and without water is not so obvious.

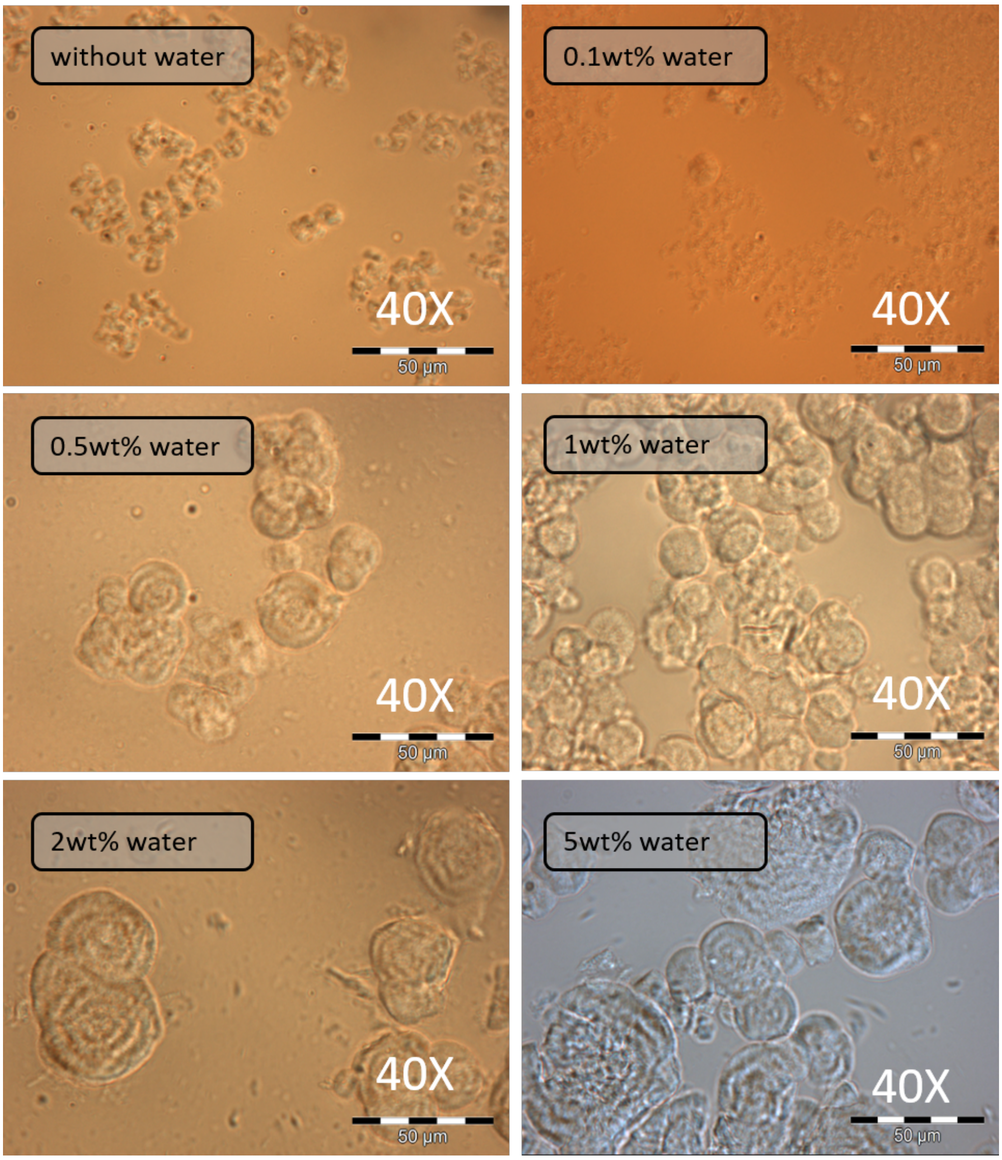

Figure S30. Variation in particle size upon regeneration of MCC from [P_{4441}][OAc]: DMSO (70:30 w/w) with different water contents (0 wt%, 0.1 wt%, 0.5 wt%, 1 wt%, 2 wt%, 5 wt%).

S13. Transmittance measurement by UV-spectrophotometer

The cloud point temperature *(Tcp)* is detected by means of transmittance measurements using a Jasco V-750 UV-Vis spectrophotometer. Transmittance was measured in the range of 90-10 °C with a cooling rate of 1 °C/min, at a wavelength of 600 nm. The concentrations of the examined cellulose solutions were varied from 7 wt% to 9.5 wt%, with an increase of 0.5 wt% per step. The cloud points were determined by fitting, as shown in Figure S31.

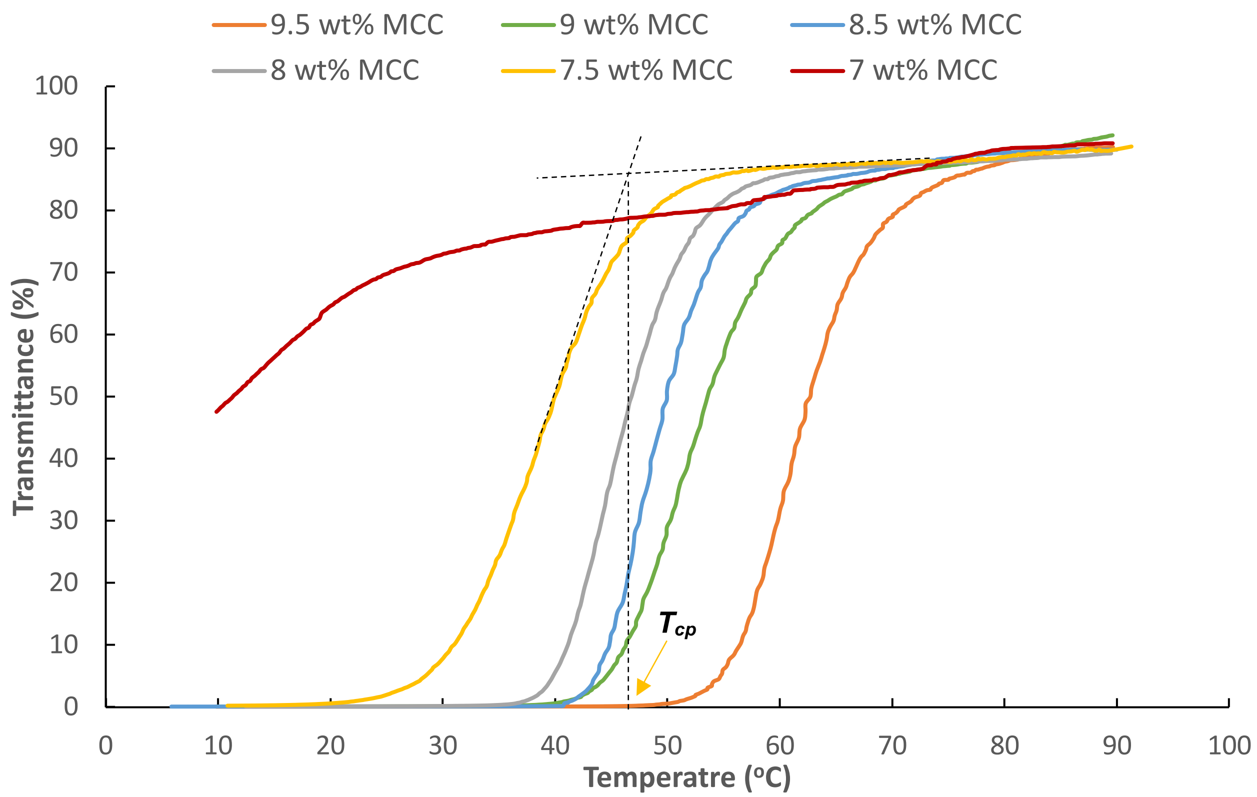

Figure S31. Change in turbidity of cellulose in [P_{4441}][OAc]:GVL (70:30 w/w) upon cooling with a constant 1 °C/min cooling rate. Example of the T_{cp} determination for the 7.5 wt.% solution is shown with dotted lines. T_{cp} for solutions with concentrations below 7.5 wt.% cannot be accurately determined.
